# lncRNA IGF2‐AS regulates miR‐500a‐3p/PPP4R1/p‐VEGFR2 signalling pathway to promote thyroid carcinoma progression and tubulogenesis

**DOI:** 10.1002/ctm2.1240

**Published:** 2023-04-17

**Authors:** Yuan Xiang, Qi‐Bei Zong, Hui Liu, Han‐Ning Li, Qi‐Fang Wu, Zhou‐Tong Dai, You Huang, Chao Shen, Le‐Wei Li, Xing‐Rui Li, Xing‐Hua Liao

**Affiliations:** ^1^ Key Laboratory for Molecular Diagnosis of Hubei Province, The Central Hospital of Wuhan, Tongji Medical College Huazhong University of Science and Technology Wuhan P.R. China; ^2^ Department of Medical Laboratory The Central Hospital of Wuhan Tongji Medical College Huazhong University of Science and Technology Wuhan P. R. China; ^3^ Institute of Biology and Medicine College of Life and Health Sciences Wuhan University of Science and Technology Hubei P. R. China; ^4^ Department of Thyroid and Breast Surgery Tongji Hospital Tongji Medical College Huazhong University of Science and Technology (HUST) Wuhan Hubei P. R. China

To the Editor

Thyroid carcinoma (THCA) continues to rise globally, with an estimated 52 890 new cases and 2180 deaths in the United States in 2020.^1^ It is reported that an increasing number of long noncoding RNAs (lncRNAs) have been involved in cancer development.^2^, ^3^, ^4^ The importance of lncRNAs in cancer, including epigenetic control, DNA damage, cell cycle regulation, regulation of microRNAs, engagement in signal transduction pathways and mediation of hormone‐induced cancer, is not to be ignored. However, the lncRNAs and underlying molecular mechanisms that play a part in THCA remain largely unknown. That is why we investigated whether a lncRNA might be able to form a competitive endogenous RNA (ceRNA) network to participate in tubulogenesis and malignancies in thyroid carcinoma. Insulin‐Like Growth Factor 2 Antisense (IGF2‐AS) was discovered as an elevated lncRNA in thyroid carcinoma in the current investigation. It acted as a ceRNA for Protein Phosphatase 4 Regulatory Subunit 1 (PPP4R1) by binding to miR‐500a‐3p.

The level of expression of IGF2‐AS in the tissues of the THCA and in the adjacent normal tissues has been demonstrated (Figure 1A). Using Kaplan–Meier analysis, the clinical consequences of IGF2‐AS expression on the prognosis of thyroid cancer patients were assessed (Figure S1A). The results demonstrated that IGF2‐AS expression was related to patient survival, and then we compared the expression of different tumour stages in thyroid carcinoma (Figure S1B). We sought gene expression variations between thyroid cancer groups with low and high IGF2‐AS expression. These are the top 50 differentially expressed genes associated with IGF2‐AS expression (Figure S1C). Enrichment analysis has shown the top five biochemical pathways (Figure S1D). A quantitative reverase transcription PCR (qRT‐PCR) examination of endogenous IGF2‐AS levels provided further evidence for this trend (Figure S1E). Together, the results suggest an oncogenic role of IGF2‐AS in thyroid carcinoma.

**FIGURE 1:**
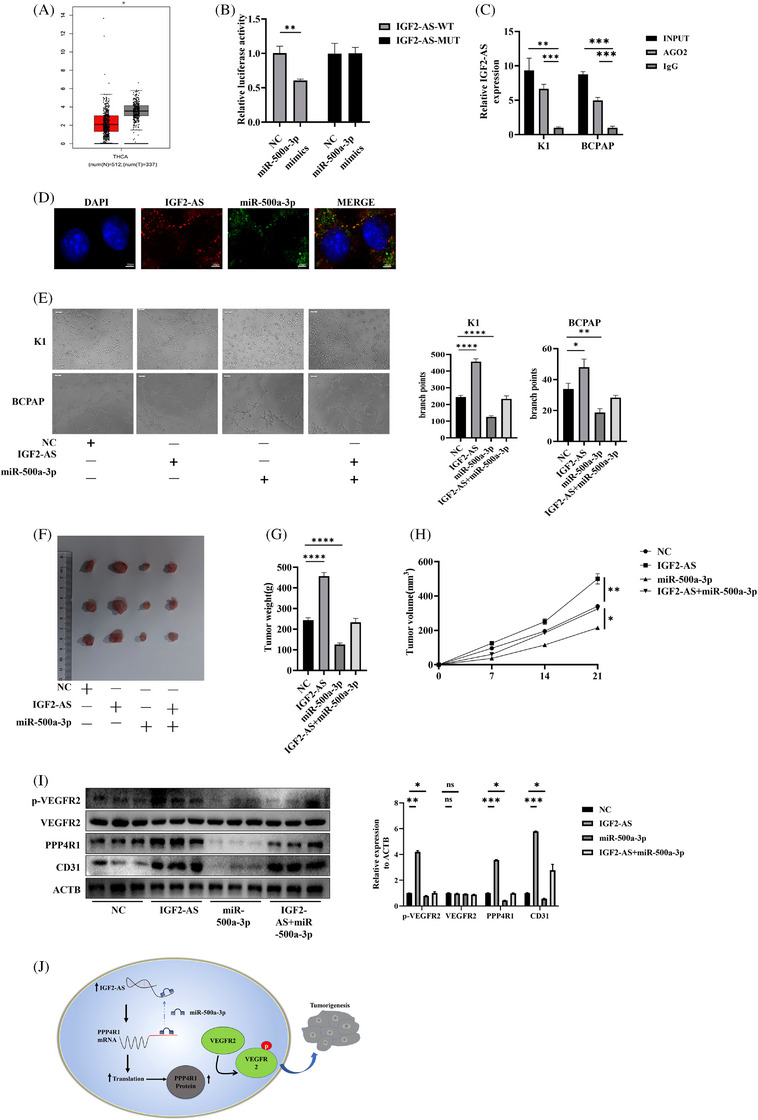
Long noncoding RNA (lncRNA)‐IGF2‐AS promotes thyroid carcinoma (THCA) via acting as a competitive endogenous RNA (ceRNA) against miR‐500a‐3p, which further upregulates PPP4R1 expression. (A) The relative expression of IGF2‐AS in THCA tumour and normal tissues from GEPIA database. (B) Luciferase assay was carried out using transfection of luciferase plasmid constructs alongside miR‐500a‐3p mimic. HEK 293T cells co‐transfected with Luc‐IGF2‐AS‐WT or Luc‐IGF2‐AS‐MUT plasmids with synthetic miR‐500a‐3p constructs. (C) Anti‐AGO2 RIP was performed to detect IGF2‐AS and miR‐500a‐3p in K1 and BCPAP cells. (D) Fluorescence in situ hybridisation (FISH) was performed with Cy3‐labelled IGF2‐AS probes (red) and FITC‐labelled miR‐500a‐3p probes (green) to detect the location of IGF2‐AS and miR‐500a‐3p in K1 cells. (E) Recovery effects of IGF2‐AS overexpression on miR‐500a‐3p overexpression‐mediated inhibition of tubulogenesis in K1 and BCPAP cells determined by tube formation assays. (F) Representative subcutaneous xenograft tumours are shown (*n* = 3). (G and H) Tumour volume and weight were measured. Data are shown as the mean ± SD based on three independent experiments. (I) The expression of PPP4R1, CD31, Vascular Endothelial Growth Factor Receptor 2 (VEGFR2) and p‐VEGFR2 in tumours as described was examined by Western blot analysis in the recovery experiments. (J) Schematic diagram of the regulatory mechanism of the IGF2‐AS/miR‐500a‐3p/PPP4R1/p‐VEGFR2 axis in promoting THCA cell tubulogenesis, proliferation, migration and invasion. Data are shown as the mean ± SD based on three independent experiments. ^*^ *p* < .05; ^**^ *p* < .01; ^***^ *p* < .001; ****p < .0001.

To investigate the biological effect of IGF2‐AS in thyroid carcinoma cells, RNA‐short hairpin RNAs (shRNAs) were used to specifically knockdown IGF2‐AS expression. By transfecting three IGF2‐AS shRNAs, the findings showed that sh‐IGF2‐AS3 could significantly downregulate IGF2‐AS (Figure S2A). Assays for tube formation and colony formation demonstrated that IGF2‐AS reduction decreased tumour tubulogenesis and migration (Figure S2B,C). Figure S2D,E discovered that depletion of IGF2‐AS impaired the tumour tubulogenesis, migration, invasion and proliferation of both K1 and BCPAP cells. Next, a xenograft mouse model was developed by injecting stable IGF2‐AS knockdown thyroid cancer cell lines (K1) subcutaneously (Figure S2F), the tumour size and weight of IGF2‐AS‐deficient animals were considerably smaller than those of control mice (Figure S2G,H). Knockdown of IGF2‐AS dramatically decreased malignant features of the tumour in vitro and tumour development in vivo, according to the findings.

Then, starBase (http://www.starbase.sysu.edu.cn/) was used to identify a miRNA termed miR‐500a‐3p as a possible IGF2‐AS‐binding regulator (Figure S3A). With TCGA database, we determined that the RNA levels of miR‐500a‐3p were substantially decreased in thyroid carcinoma patients in comparison to the control group (Figure S3B). Additionally, we discovered that high RNA levels of miR‐500a‐3p boosted the survival probability of THCA patients (Figure S3C). Additional investigation revealed a connection between declining miR‐500a‐3p levels and rising IGF2‐AS levels (Figure S3D). Next, the wild‐type (IGF2‐AS‐WT) and mutant‐type (IGF2‐AS‐MUT) miR‐500a‐3p dual‐luciferase expression binding sites in thyroid carcinoma were constructed (Figure 1B). It is considered that the localisation of lncRNA in the cell serves as a marker for identifying the regulatory mechanisms of lncRNA,^5^ and we suspected that IGF2‐AS is involved in THCA progression by ceRNA mechanism. RNA Binding Protein Immunoprecipitation Assay (RIP) experiments demonstrated that AGO2 directly binds to IGF2‐AS (Figure 1C). The results showed that IGF2‐AS and miR‐500a‐3p were mostly detected in the cytoplasm of thyroid cancer cells (Figure 1D). In contrast to IGF2‐AS, the RNA levels of miR‐500a‐3p were significantly reduced in four thyroid cancer cell lines relative to normal thyroid cells (Figure S3E). In thyroid cancer cells, the findings revealed that IGF2‐AS played the role of a sponge for miR‐500‐3p. When IGF2‐AS shRNAs were transfected into thyroid cancer cells, miR‐500a‐3p levels rose (Figure S3F). However, when miR‐500a‐3p mimics were transfected into thyroid cancer cells, IGF2‐AS was downregulated (Figure S3G).

Tube formation, colony formation assays, wound healing and transwell assays were promoted by IGF2‐AS overexpression; this impact might be partly reversed by overexpression of miR‐500‐3p (Figures 1E and S4A–C). Nude mice were induced to co‐express miR‐500a‐3p and IGF2‐AS by subcutaneous injection. IGF2‐AS overexpression substantially accelerated the development of subcutaneous tumours of thyroid carcinoma cells, but miR‐500a‐3p overexpression inhibited the IGF2‐AS‐enhanced induced tumourigenicity in vivo (Figure 1F–H). The findings demonstrated that IGF2‐AS overexpression increased in vitro and in vivo cell growth and proliferation by controlling miR‐500a‐3p. To further examine the involvement of miR‐500a‐3p in THCA, K1 and BCPAP cells were transfected with negative control (NC) or a miR‐500a‐3p mimic. Overexpression of miR‐500a‐3p decreased cell tubulogenesis, proliferation, migration and invasion, according to our results (Figure S5A–D). These results give further evidence for the functional significance of miR‐500a‐3p in thyroid cancer cell responses.

To determine if mRNA turnover is a feasible strategy for regulating PPP4R1 protein levels, miR‐500a‐3p was detected after bioinformatic analysis (Figure S6A,B). To evaluate the relationship between miR‐500a‐3p, PPP4R1 and cancer status, PPP4R1 levels were found to be considerably elevated in thyroid cancer patients compared to controls (Figure S6C). In particular, decreased miR‐500a‐3p expression was associated with a poor outcome in THCA patients in TCGA database (Figure S6D). Further investigation revealed a connection between decreasing levels of miR‐500a‐3p and rising levels of PPP4R1 (Figure S6E). The qRT‐PCR and Western blot data demonstrated that PPP4R1 was upregulated in all THCA cell lines investigated (Figure S6F,G). Using a dual‐luciferase reporter assay, the luciferase cDNA was fused to either the wild‐type PPP4R1 3′UTR (PPP4R1‐WT) or a mutant‐type PPP4R1 3′UTR (PPP4R1‐MUT) that was incapable of binding miR‐500a‐3p (Figure S6H). PPP4R1 expression was reduced by qRT‐PCR analysis of RNA levels in thyroid cancer K1 and BCPAP cells transfected with a miR‐500a‐3p mimic (Figure S6I).

PPP4R1 shRNAs were developed to evaluate the biological involvement of PPP4R1 in thyroid cancer. qRT‐PCR tests validated the knockdown's effectiveness (Figure S7A). PPP4R1 mRNA levels were considerably lower in shRNA‐infected K1 and BCPAP cells than in NC cells (Figure S7B). The protein levels of PPP4R1, VEGFR2 and p‐VEGFR2 in cells suggested that VEGFR2 phosphorylation is reduced when PPP4R1 is inhibited (Figure S7C). Tube formation, colony formation assays, wound healing and transwell assays showed that PPP4R1 inhibition decreases cell tubulogenesis, proliferation, migration and invasion (Figure S7D–G). In conclusion, PPP4R1 expression levels affect cell tubulogenesis, proliferation, migration and invasion.

We further determined whether PPP4R1 regulated tumourigenicity of thyroid carcinoma cells using generation of xenograft. Tumours collected from mice were exhibited and measured (Figure S8A–C). Moreover, the PPP4R1 mRNA expression of the sh‐PPP4R1 group was significantly decreased in tumours compared with sh‐NC treatment group (Figure S8D). Haematoxylin and eosin staining revealed that reduction of PPP4R1 greatly decreased the tumour's malignancy (Figure S8E). In addition, PPP4R1, p‐VEGFR2 and IGF2‐AS expression levels were positively associated (Figure S8F,G). In conclusion, the present findings reveal that PPP4R1 might be a therapeutic target for THCA.

Next, Western blotting and immunohistochemistry (IHC) were used to detect PPP4R1, CD31 and p‐VEGFR2 protein levels in tumours from nude mice, and the results showed that significant reductions in CD31, PPP4R1 and p‐VEGFR2 were seen in the sh‐IGF2‐AS group (Figure S9A–C). Subsequently, we used Western blotting and IHC to detect PPP4R1, CD31 and p‐VEGFR2 in tumours after overexpression of miR‐500a‐3p and IGF2‐AS in nude mice. The results showed that PPP4R1, CD31 and p‐VEGFR2 were significantly increased by upregulating IGF2‐AS; these effects could be reversed by an increase in miR‐500a‐3p levels (Figures 1I and S9D,E). These findings revealed that IGF2‐AS/miR‐500a‐3p/PPP4R1 stimulated the advancement of tubulogenesis in thyroid cancer cells by activating the VEGFR2 pathway (Figure 1J).

In summary, our study showed that IGF2‐AS acts as an oncogenic lncRNA in THCA. IGF2‐AS/miR‐500a‐3p/PPP4R1/p‐VEGFR2 axis could be the future of thyroid treatment.

## CONFLICT OF INTEREST STATEMENT

The authors declare no conflicts of interest.

## Supporting information

Supporting Information

Supporting Information

Supporting Information

Supporting Information

Supporting Information

Supporting Information

Supporting Information

Supporting Information

Supporting Information

Supporting Information

Supporting Information

Supporting Information

Supporting Information
