# Supplementary material for: lncRNA IGF2‐AS regulates miR‐500a‐3p/PPP4R1/p‐VEGFR2 signalling pathway to promote thyroid carcinoma progression and tubulogenesis
Source: Clin Transl Med. 2023 Apr 17;13(4):e1240. doi: 10.1002/ctm2.1240 (PMC10111635; doi:10.1002/ctm2.1240)
Supplement: Supplementary file 1 — Supporting Information [file CTM2-13-e1240-s003.docx]

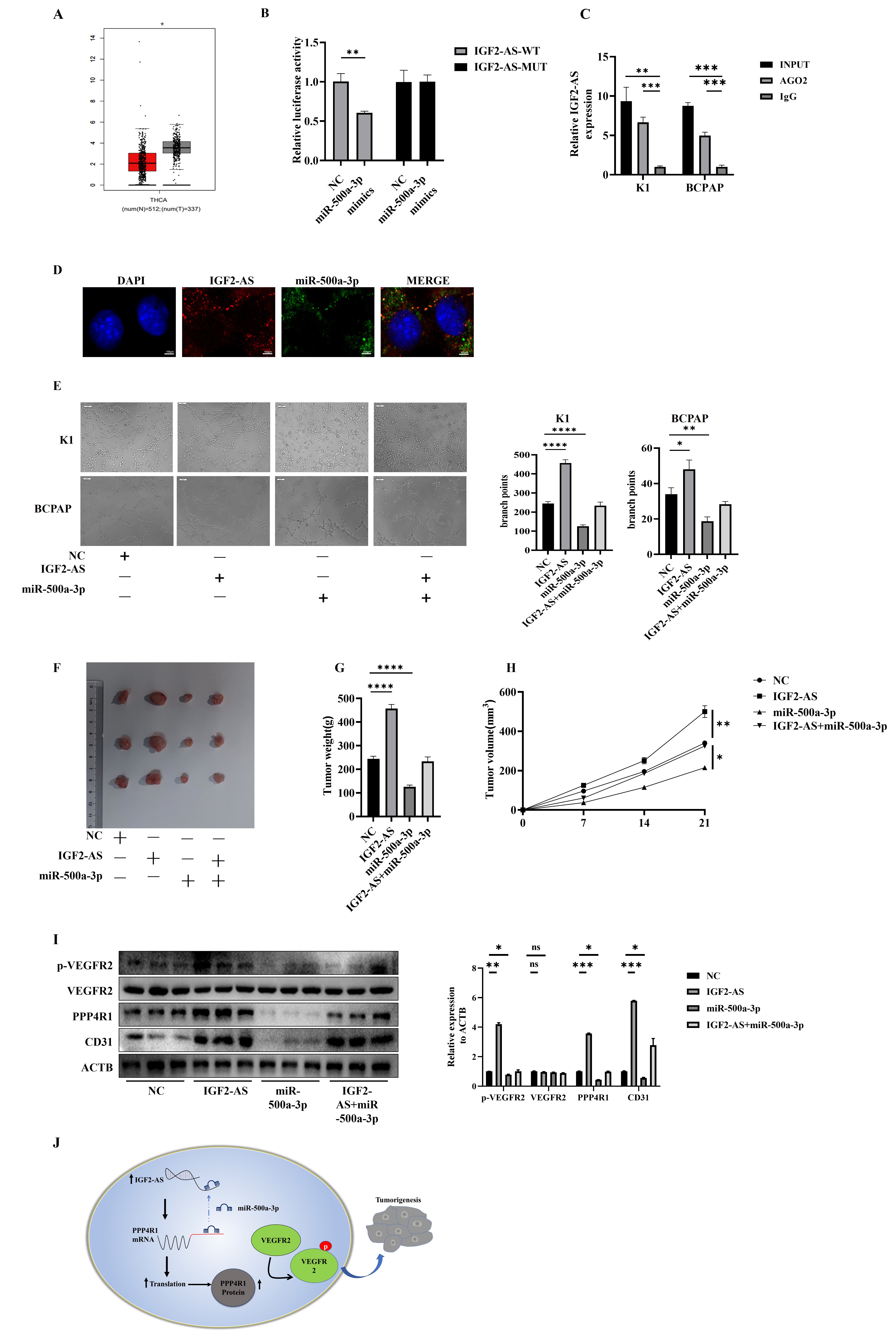


# Fig. 1 IGF2-AS was upregulated in thyroid cancer tissues and cells. (A) The correlation between prognosis and the expression of IGF2-AS in THCA patients from Starbase database. (B) Relative IGF2-AS expression in lung cancer tissues with different TNM stages. (C) The top 50 differentially expressed genes (linked to IGF2-AS overexpression) in thyroid cancer disease presented as a gene expression heat map. (D) Kyoto Encyclopedia of Genes and Genomes (KEGG) biochemical pathway enrichment analysis; adjusted p<0.05. (E) Analysis of relative IGF2-AS levels in Nthy-ori 3-1, TPC-1, BCPAP and K1 thyroid cell lines using qRT-PCR. Data are shown as the mean ± SD based on three independent experiments. *P<0.05; **P<0.01; ***P<0.001; ****P<0.0001.


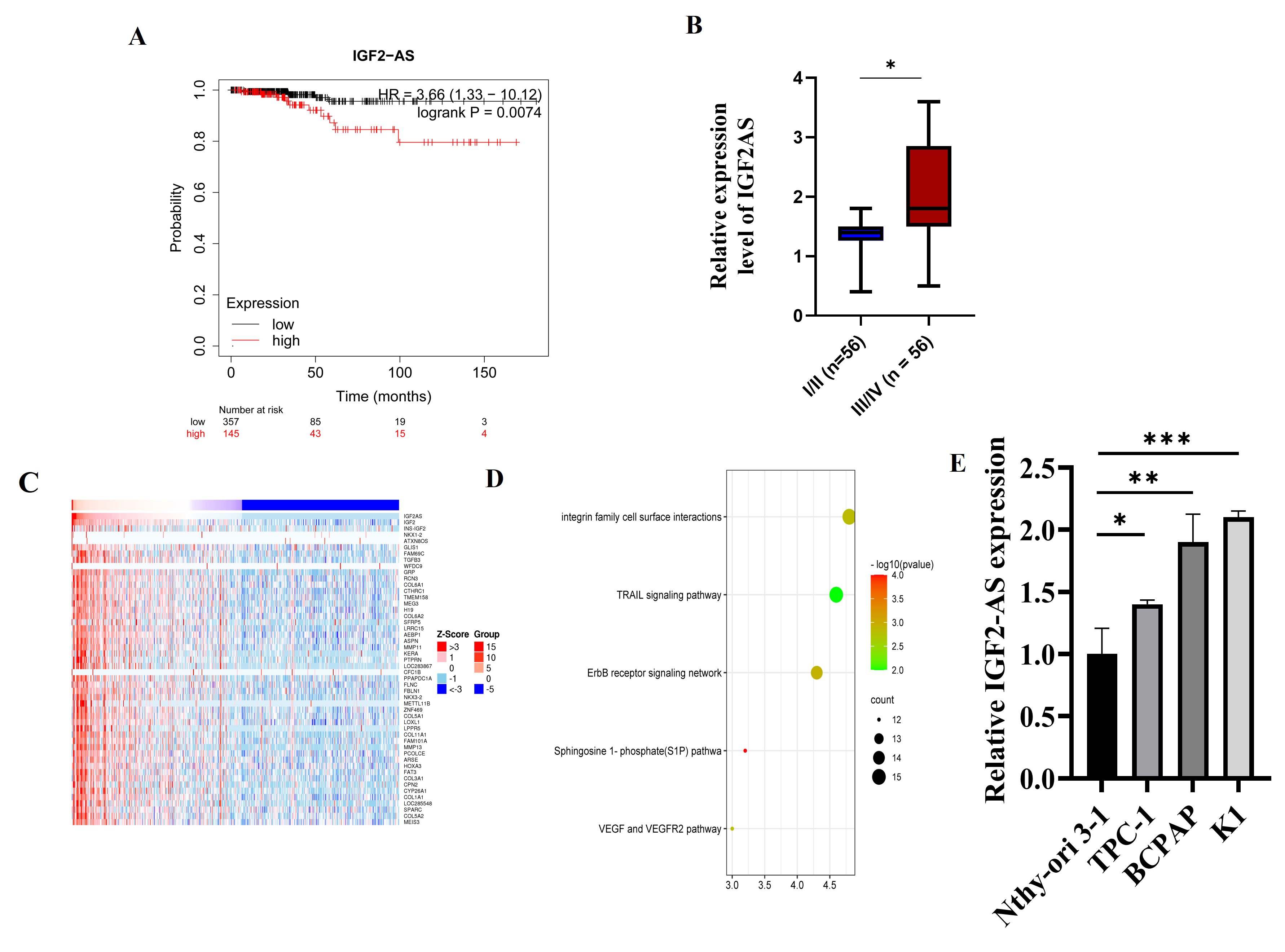


**Fig. S1** IGF2-AS was upregulated in thyroid cancer tissues and cells. (**A**) The relative expression of IGF2-AS in THCA tumor and normal tissues from GEPIA database. (**B**) The correlation between prognosis and the expression of IGF2-AS in THCA patients from Starbase database. (**C**) Relative IGF2-AS expression in lung cancer tissues with different TNM stages. (**D**) The top 50 differentially expressed genes (linked to IGF2-AS overexpression) in thyroid cancer disease presented as a gene expression heat map. (**E**) Kyoto Encyclopedia of Genes and Genomes (KEGG) biochemical pathway enrichment analysis; adjusted p<0.05. (**F**) Analysis of relative IGF2-AS levels in Nthy-ori 3-1, TPC-1, BCPAP and K1 thyroid cell lines using qRT-PCR. Data are shown as the mean ± SD based on three independent experiments. *P<0.05; **P<0.01; ***P<0.001**;** ********P<0.0**0**01.


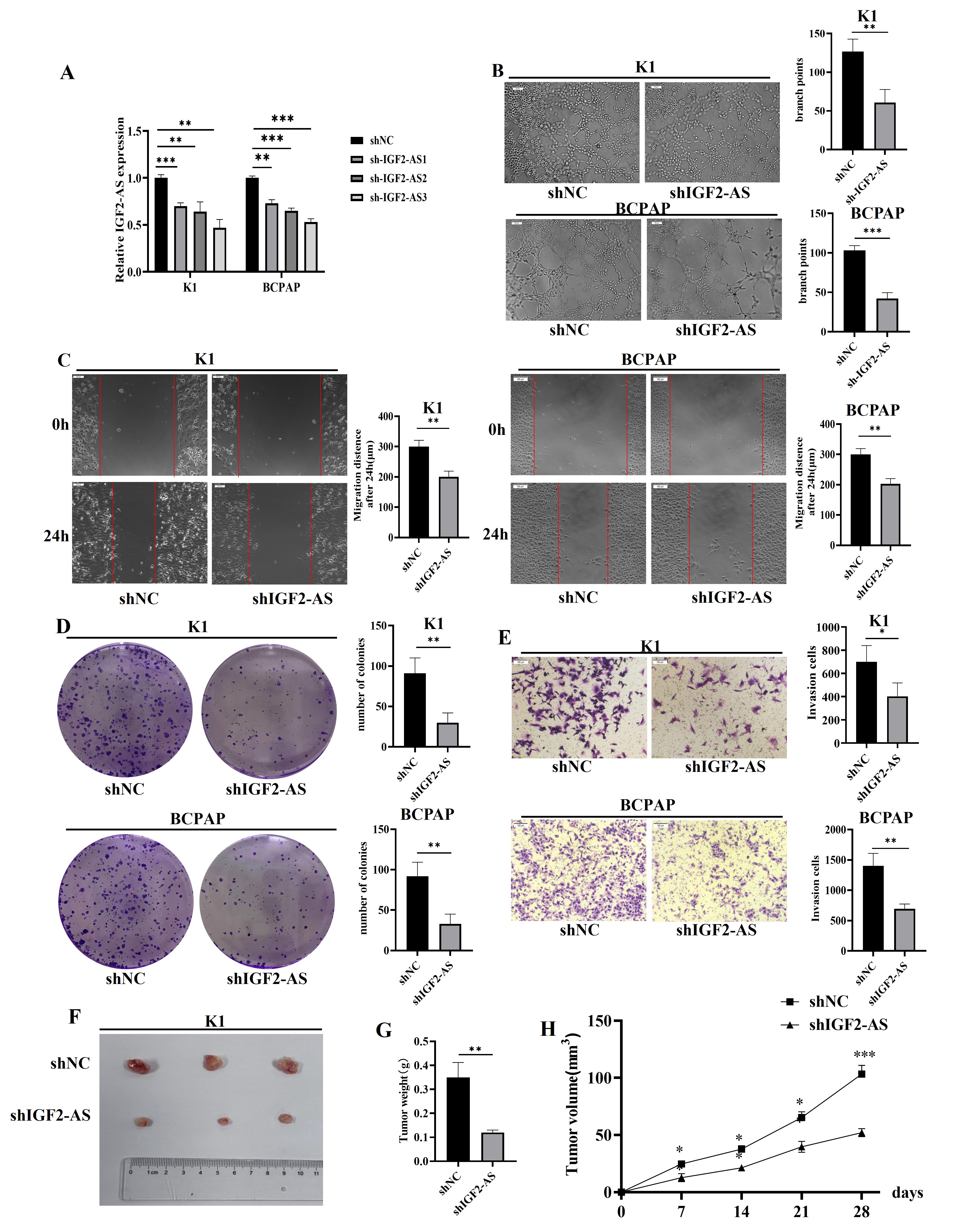


**Fig.S2** IGF2-AS promotes thyroid cell tubulogenesis, proliferation, colony formation, migration, and invasion in vitro and tumor growth in vivo. **(A)** K1 cells and BCPAP cells were transfected with negative control shRNA (shNC) or three independent shRNAs targeting IGF2-AS (shIGF2-AS1, shIGF2-AS2 and shIGF2-AS3) followed by RT-qPCR analysis. **(B)** The tubulogenesis ability of K1 and BCPAP cells treated with shIGF2-AS3 was assessed by tube formation assays. The bar charts represent the branch points of the cell tubulogenesis. **(C)** The migration ability of K1 and BCPAP cells treated with sh-IGF2-AS1 was assessed by wound healing assays. The bar charts represent the distance of the cell migration. **(D)** Colony formation assays were used to detect the proliferation of thyroid cancer cells after transfection with shIGF2-AS in K1 and BCPAP cells. The bar charts represent the numbers of cell colonies. **(E)** The invasion ability of K1 and BCPAP cells treated with sh-IGF2-AS was evaluated by the transwell assays. The bar charts indicate the number of invaded cells. **(F)** Representative subcutaneous xenograft tumors were shown (n = 3). **(G,H)** Tumor volume and weight in the xenograft mice from the IGF2-AS knockdown group and the control group. Data are shown as the mean ± SD in three independent experiments. *P<0.05; **P<0.01; ***P<0.001**;** ********P<0.0**0**01.


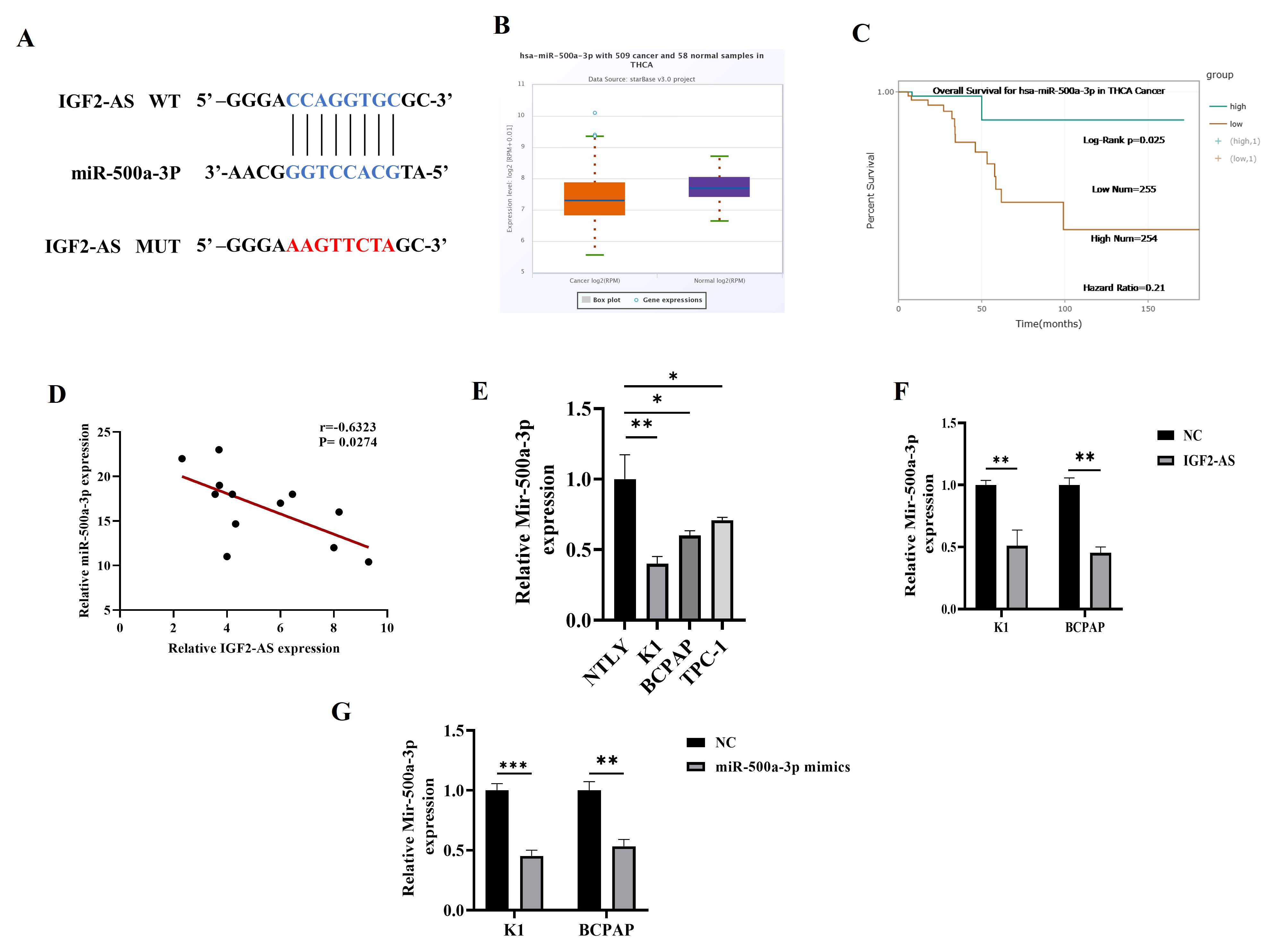


**Fig.S3** IGF2-AS acted as a sponge for miR-500a-3p . **(A)** Schematic depiction of the predicted binding site for miR-500a-3p in the IGF2-AS. **(B)** Analysis of miR-500a-3p levels in Thyroid cancer tumors vs. normal tissues in the THCA database in TCGA. **(C)** The correlation between the expression of miR-500a-3p and prognosis of THCA patients in house(n=509). **(D)** Correlative analysis of miR-500a-3p and IGF2-AS levels in Thyroid cancer tumors using the THCA database. **(E)** Profiling of miR-500a-3p levels in normal and cancer Thyroid cancer cell lines using qRT-PCR. Data are shown as the mean ± SD based on three independent experiments. **(F)** Relative miR-500a-3p expression after IGF2-AS knockdown. **(G)** Relative miR-500a-3p expression after transfected miR-500a-3p mimic. *P<0.05; **P<0.01; ***P<0.001**;** ********P<0.0**0**01.


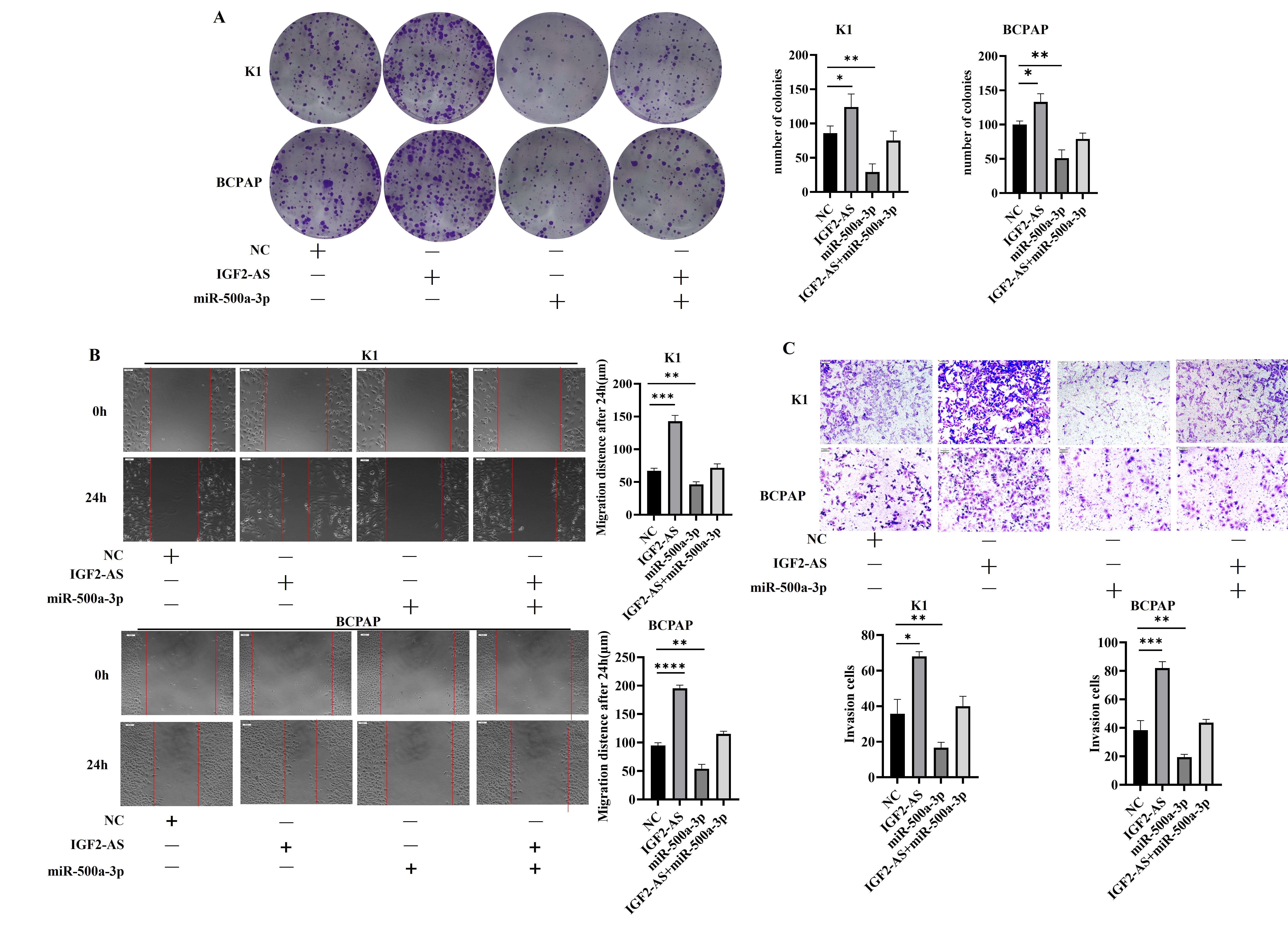


**Fig.S4** IGF2-AS overexpression promoted cell growth and proliferation in vitro and in vivo by regulating miR-500a-3p. **(A)** Rescue effects of IGF2-AS overexpression on miR-500a-3p overexpression-mediated inhibition of cell growth in K1 and BCPAP cells determined by colony formation assays. **(B)** Rescue effects of IGF2-AS overexpression on miR-500a-3p overexpression-mediated inhibition of cell migration in K1 and BCPAP cells determined by wound healing assays. **(C)** Rescue effects of IGF2-AS overexpression on miR-500a-3p overexpression-mediated inhibition of cell invasion in K1 and BCPAP cells determined by transwell assays. *P<0.05; **P<0.01; ***P<0.001**;** ********P<0.0**0**01.


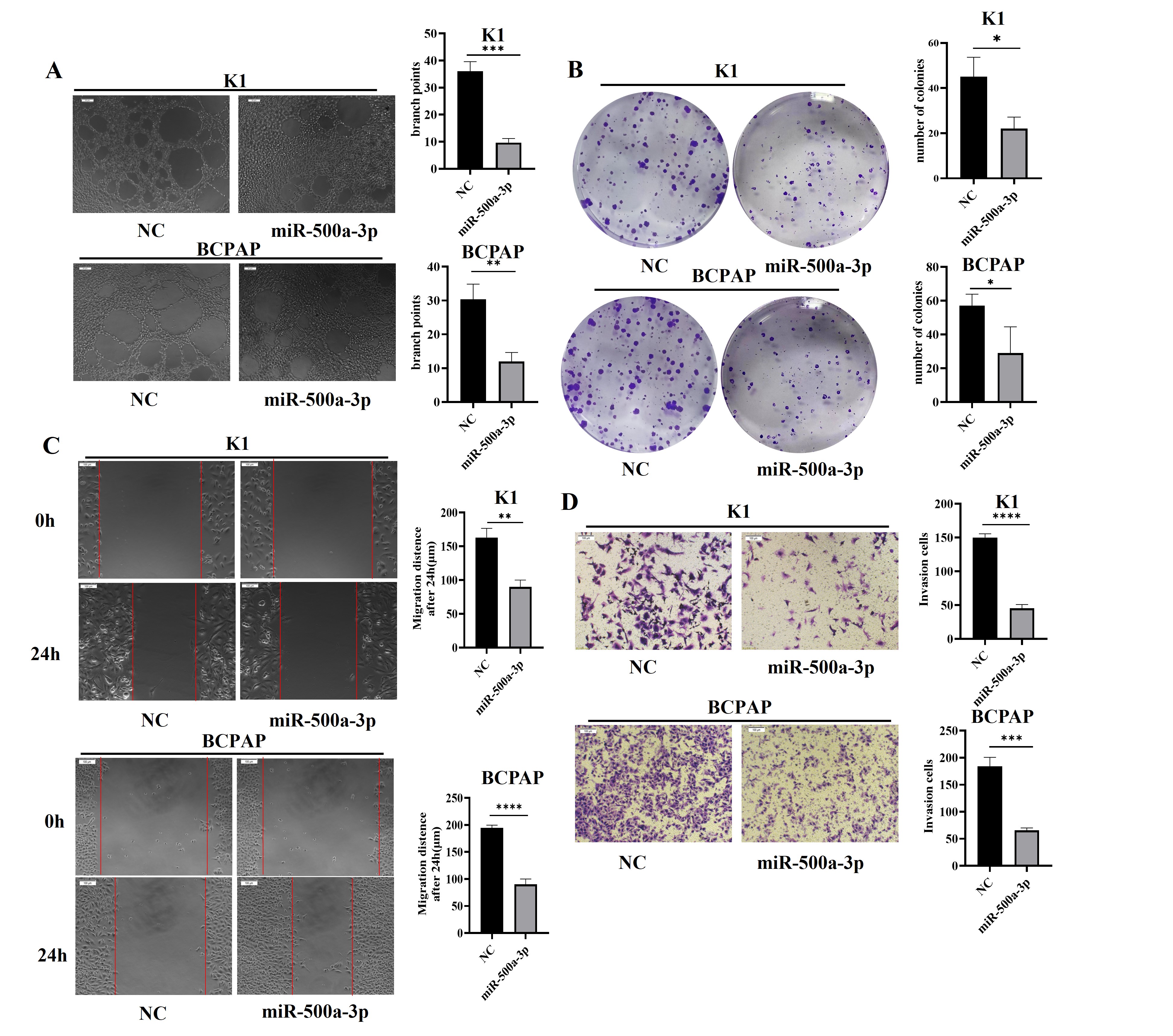


**Fig.S5** miR-500a-3p effects on thyroid cancer cell responses. **(A)** The tubulogenesis ability of K1 and BCPAP cells treated miR-500a-3p overexpression was assessed by tube formation assays .The bar charts represent the branch points of the cell tubulogenesis. **(B)**Colony formation assays were used to detect the proliferation of thyroid cancer cells after transfection with miR-500a-3p overexpression in K1 and BCPAP cells. The bar charts represent the numbers of cell colonies. **(C)** The migration ability of K1 and BCPAP cells treated with miR-500a-3p overexpression was assessed by wound healing assays. The bar charts represent the distance of the cell migration. **(D)** The invasion ability of K1 and BCPAP cells treated with miR-500a-3p overexpression was evaluated by the transwell assays. The bar charts indicate the number of invaded cells. Data are shown as the mean ± SD based on three independent experiments. *P<0.05; **P<0.01; ***P<0.001**;** ********P<0.0**0**01.


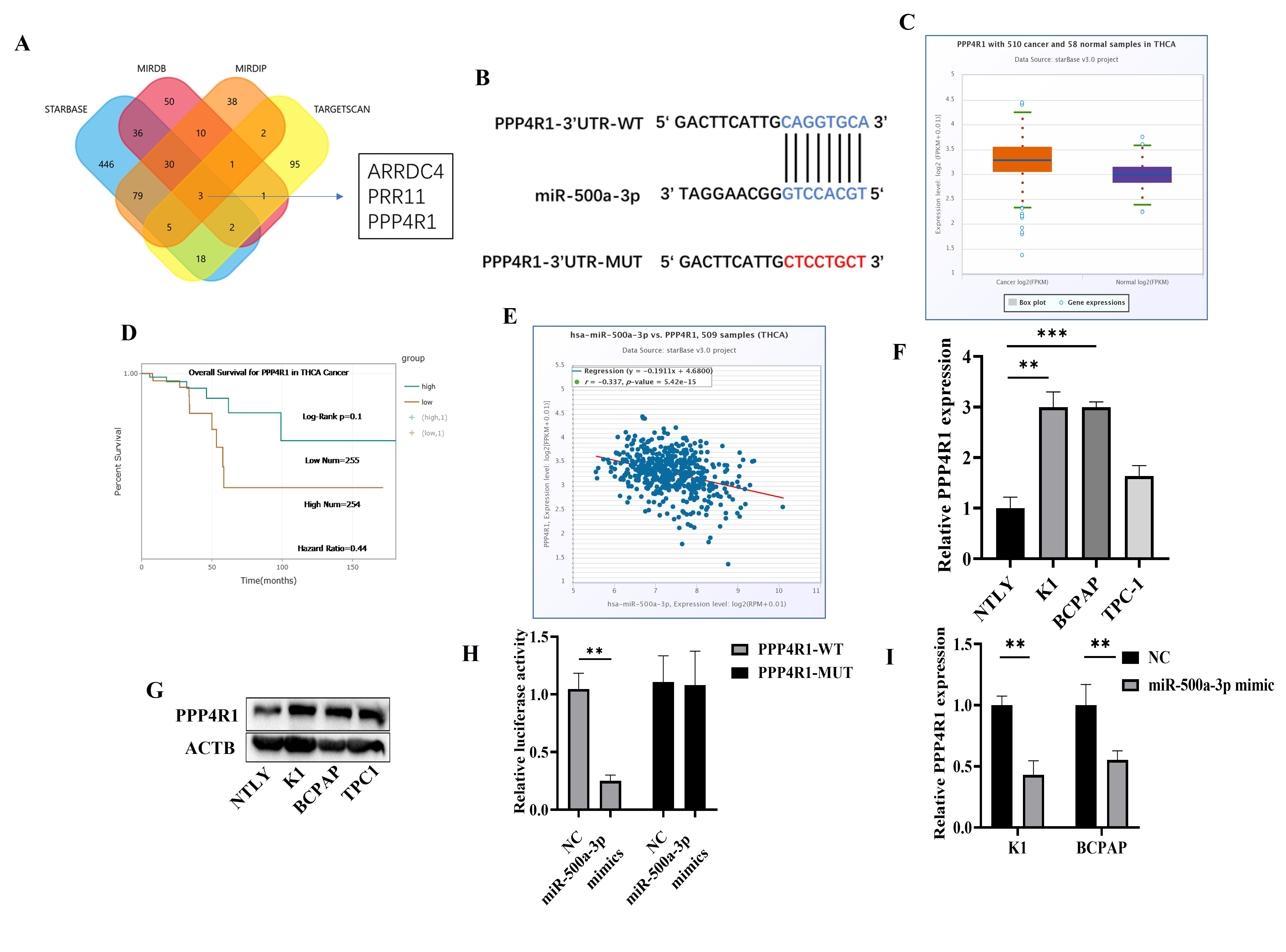


**Fig.S6** PPP4R1 mRNA is a target of miR-500a-3p. **(A)** Venn diagram showing miR-500a-3p target gene from the results of TATGETSCAN,MIRDIP,MIRDB and Starbase database prediction. **(B)** Schematic depiction of the predicted binding site for miR-500a-3p in the PPP4R1. **(C)** Analysis of PPP4R1 levels in Thyroid cancer tumors vs. normal tissues in the THCA database in TCGA. **(D)** The correlation between prognosis and the expression of PPP4R1 in THCA patients from Starbase database. **(E)** Correlative analysis of miR-500a-3p and PPP4R1 levels in Thyroid cancer tumors using the THCA database. **(F,G)** Profiling of PPP4R1 levels in normal and cancer Thyroid cancer cell lines using qRT-PCR and Western blot. **(H)** Relative luciferase activities of wild type (WT) and mutated (MUT) PPP4R1 reporter plasmid co-tr ansfected with miR-500a-3p mimics in 293T cells. **(I)** Relative PPP4R1 expression after transfection with miR-500a-3p mimic in K1 and BCPAP cell line. Data are shown as the mean ± SD based on three independent experiments. *P<0.05; **P<0.01; ***P<0.001**;** ********P<0.0**0**01.


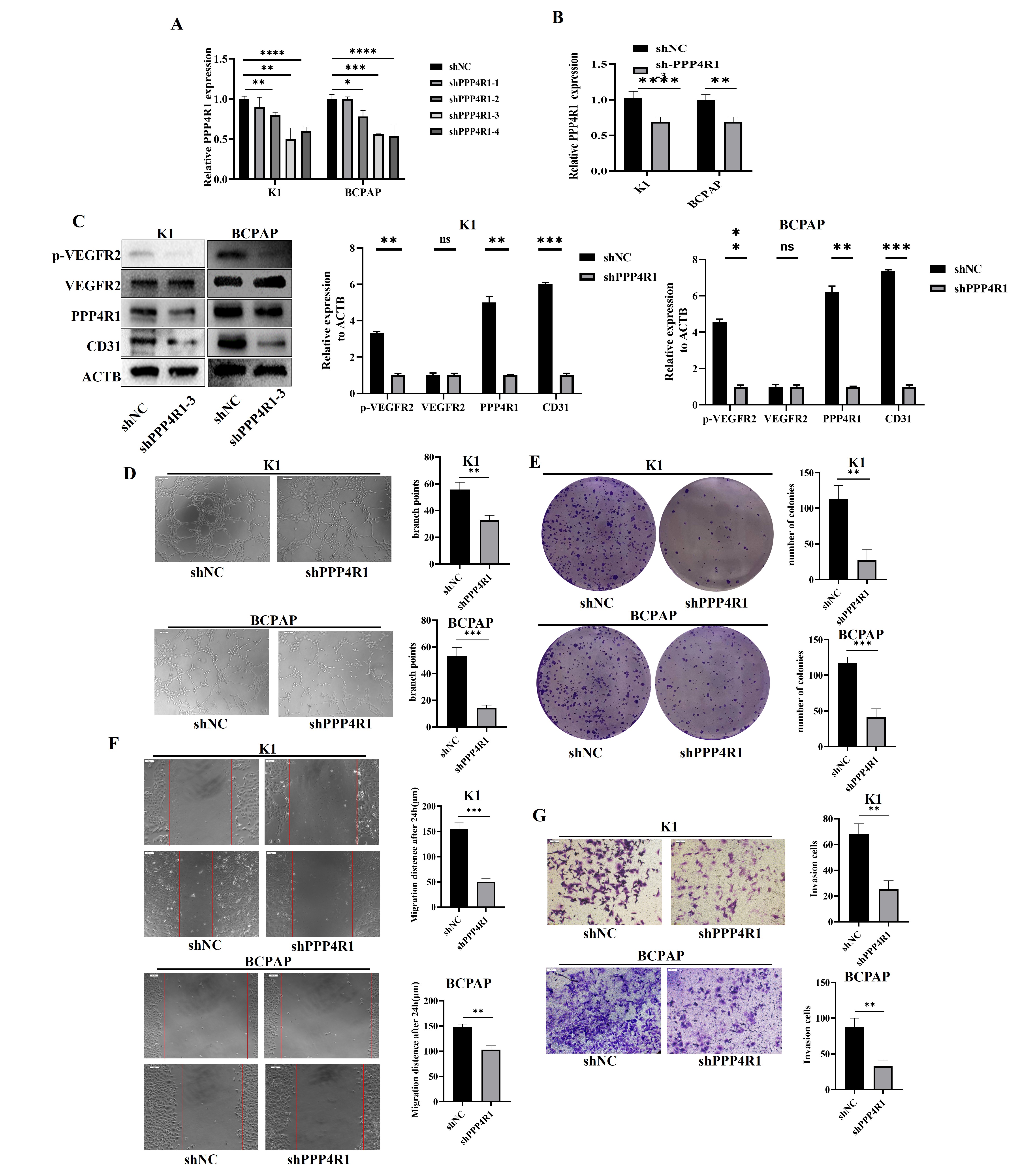


**Fig.S7** Knockdown of PPP4R1 inhibits tumorigenicity of Thyroid cancer cells. **(A)** PPP4R1 knockdown in Thyroid cancer cell line K1 and BCPAP monitored by qRT-PCR. **(B)** Relative PPP4R1 expression transfection with shPPP4R1-3. **(C)**Western blot analysis for PPP4R1 in K1 and BCPAP cells after transfection with shPPP4R1-3. **(D)** The tubulogenesis ability of K1 and BCPAP cells treated with shPPP4R1 was assessed by tube formation assays .The bar charts represent the branch points of the cell tubulogenesis . **(E)** The migration ability of K1 and BCPAP cells treated with shPPP4R1 was assessed by wound healing assays. The bar charts represent the distance of the cell migration. **(F)** Colony formation assays were used to detect the proliferation of thyroid cancer cells after transfection with shPPP4R1 in K1 and BCPAP cells. The bar charts represent the numbers of cell colonies. **(G)** The invasion ability of K1 and BCPAP cells treated with shPPP4R1 was evaluated by the transwell assays. The bar charts indicate the number of invaded cells. Data are shown as the mean ± SD based on three independent experiments. *P<0.05; **P<0.01; ***P<0.001**;** ********P<0.0**0**01.


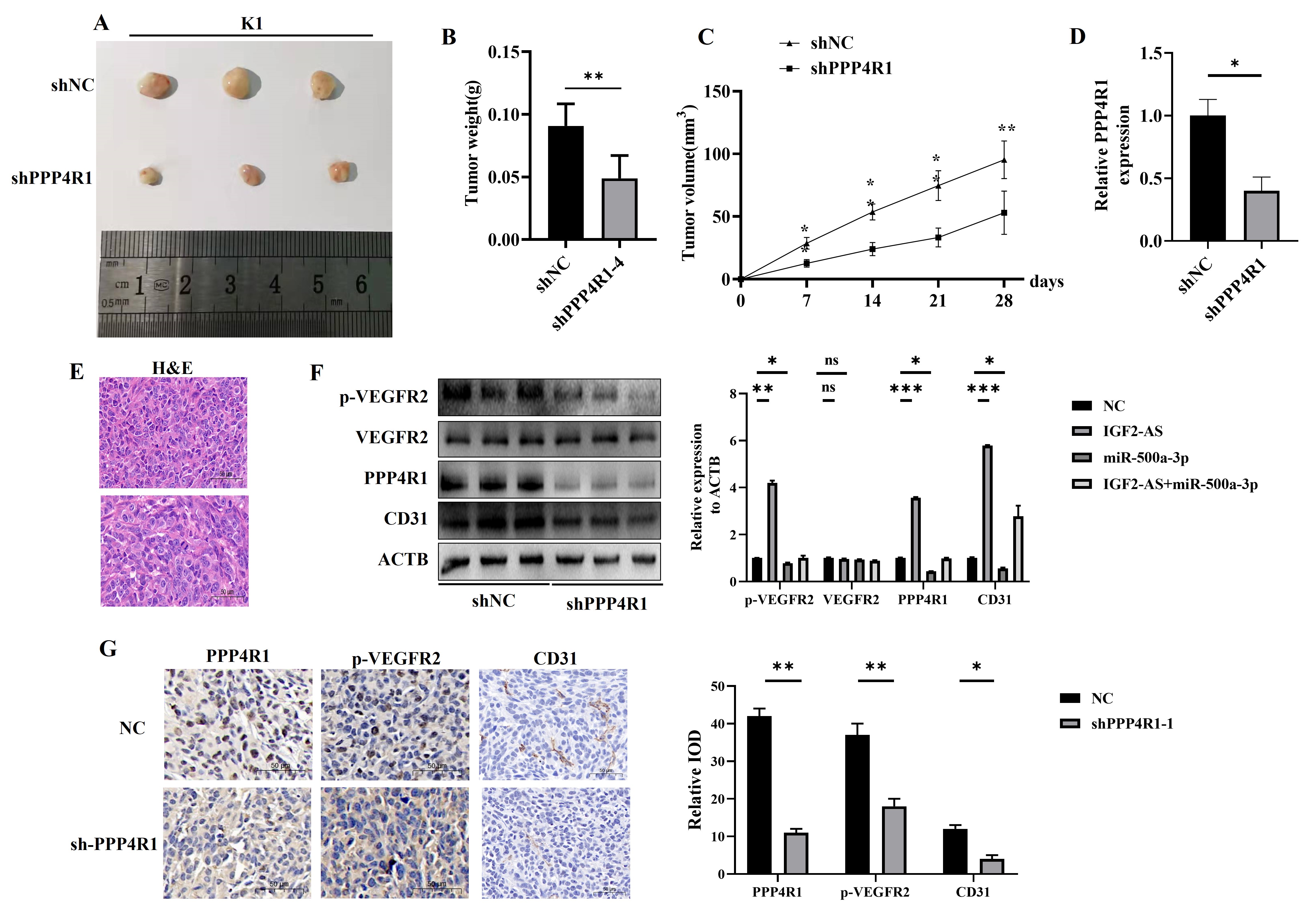


**Fig.S8** Knockdown of PPP4R1 inhibits tumorigenicity of Thyroid cancer cells. **(A)** Excision and viewing of subcutaneous tumors derived from Nude mice injected for control K1 cells (NC) or cells expressing siRNA specific for PPP4R1 (shPPP4R1). **(B,C)** Analysis of weight or volume of tumors derived from Nude mice injected for control K1 cells (NC) or cells expressing siRNA-specific for PPP4R1 (shPPP4R1). **(D)** Analysis of PPP4R1 using RT-qPCR of tumors from Nude mice injected for control K1 cells (NC) or cells expressing siRNA-specific for PPP4R1 (shPPP4R1). **(E)** Analysis of p-VEGFR2,VEGFR2,PPP4R1 and CD31 using Western blotting of tumors from nude mice injected for control K1 cells (NC) or cells expressing siRNA-specific for PPP4R1 (shPPP4R1). **(F)** Hematoxylin-eosin on tissue sections from subcutaneous tumors derived from Nude mice injected for control K1 (NC) or cells expressing siRNA-specific for PPP4R1 (shPPP4R1). **(G)** PPP4R1,p-VEGFR2,CD31 immunohistochemistry on tissue sections from subcutaneous tumors derived from Nude mice injected for control K1cells (NC) or cells expressing shRNA-specific for PPP4R1 (shPPP4R1-1). Data are shown as the mean ± SD based on three independent experiments. *P<0.05; **P<0.01; ***P<0.001**;** ********P<0.0**0**01.


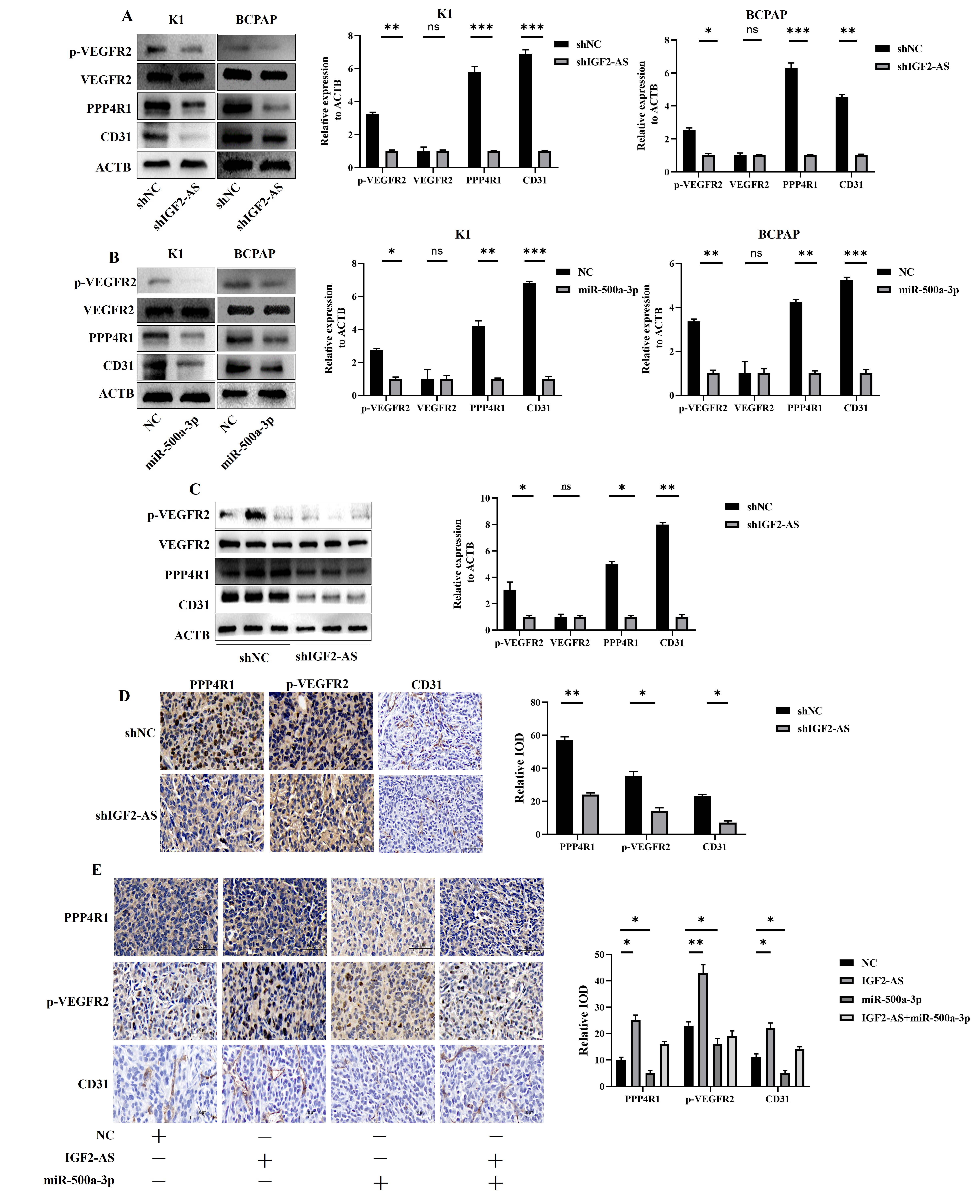


**Fig.S9** IGF2-AS/miR-500a-3p/PPP4R1 promoted tubulogenesis progression in thyroid cancer cells by activating the VEGFR2 pathway. **(A)** Western blot analysis for PPP4R1,CD31,VEGFR2,p-VEGFR2 in K1 and BCPAP cells after transfection with sh-IGF2-AS. **(B)** Western blotting was conducted to verify the protein expression of PPP4R1,CD31,VEGFR2,p-VEGFR2 in K1 and BCPAP cells after miR-500a-3p overexpression. **(C)** The expression of IGF2-AS in tumors as described was examined by Western blot analysis. **(D)** Representative IHC staining of PPP4R1, p-VEGFR2 and CD31 in the tumors from nude mice with subcutaneous implantation in the experiments. **(E)** Representative IHC staining of PPP4R1, p-VEGFR2 and CD31 in the tumors from nude mice with subcutaneous implantation in the rescue experiments
