# Supplementary material for: lncRNA IGF2‐AS regulates miR‐500a‐3p/PPP4R1/p‐VEGFR2 signalling pathway to promote thyroid carcinoma progression and tubulogenesis
Source: Clin Transl Med. 2023 Apr 17;13(4):e1240. doi: 10.1002/ctm2.1240 (PMC10111635; doi:10.1002/ctm2.1240)
Supplement: Supplementary file 2 — Supporting Information [file CTM2-13-e1240-s008.docx]

**MATERIALS AND METHODS**

**Tissue samples and cell lines**

Tissue samples and cell lines THCA tissue samples and normal Thyroid carcinoma tissue samples were collected from the Tongji Hospital (Wuhan, P. R. China) in the time of 2021.10 to 2022.3. This study obtained the informed consent of all patients and the approval of the Ethics Committee of the Cancer Hospital of Hubei. Nthy-ori 3-1, K1, BCPAP and TPC-1 cell lines were obtained from the Cell Bank of the Chinese Academy of Sciences (Shanghai, China).

**Transfection**

THCA cells were transfected with shRNAs, miRNAs, or plasmids using Lipofectamine 2000 (Invitrogen). ShIGF2-AS and shPPPP4R1 were obtained from TsingkeBio (Beijing, China). miRNA mimics was purchased from RiboBio (GuangZhou, China). The human PPPP4R1 overexpression plasmid PPPP4R1-bio-His was purchased from Addgene (Cambridge, MA).

**Real-time RT-PCR**

RNA was extracted using TRIzol reagent (TaKaRa, Japan). 2 ug RNA was reverse-transcribed into cDNA with MLV-reverse transcriptase (Invitrogen), and Hieff qRT-PCR SYBR Green Master Mix was used for qRT-PCR (Tiangen, China). The primer sequences were as follows, IGF2-AS: 5′-TTHCAACCCCTHCATTGCACACG-3′ and 5′-CTGGCTGTTHCAAGAATTGAGGTAA-3′; miR-500a-3p: 5'-ATGCACCTGGGCAAGGATTHCAT-3' and 5'-AGTGCAGGGTHCACGAGGTATTHCA-3'; U6: 5'-CGAATTTGCGTGTHCAATHCACT-3' and 5'-CGCTTHCAGGCAGCACATATA-3’; PPPP4R1: 5'-CACAAGATGAAATGTTGACGCC-3' and 5'- CAAGGTATHCAGAGCAAACTHCACG-3'; GAPDH: 5'- ATGACATHCAAA GAAGGTGGTG -3' and 5'- CATACCAGGAAATGA GCTTG -3'.

**Western blotting**

THCA cells were lysed by RIPA buffer for 20min at 0° C. The proteins were processed by SDS-PAGE and transferred to PVDF membrane (BioRad). The primary antibodies used in Western blotting were as follows: anti-PPPP4R1 (A8361, 1：2000 dilution, ABclonal), anti-VEGFR2 (26415-1-AP, 1：1000 dilution, Proteintec), anti-p-VEGFR2 (67392-1-Ig, 1:500 dilution, ABclonal), anti-CD31 (11265-1-AP, 1:1000 dilution, Proteintec).

**RNA immunoprecipitation (RIP)**

RNA-protein-antibody complexes were captured using Protein A/G (ThermoFisher, USA). RNA was eluted by adding TRIzol directly to magnetic beads and isolated as per the manufacturer's instructions. cDNA was synthesized using HiScript® II 1st Strand cDNA Synthesis Kit (Vazyme, China) and analyzed by qRT-PCR.

**Tube formation assay**

Matrigel (BD Biosciences) was used to observed tube formation of primary HUVECs. Then, 1×10^4^ primary HUVECs in 50 µl of conditioned medium derived from K1 and BCPAP cells were seeded. At 6 h after seeding, images were collected using light microscopy. The total tube length was measured and analyzed with Image-Pro-Plus 6.0.

**Colony formation assay**

The transfected cell suspension was collected, and 500 cells were seeded into a 6-well plate and cultured in a cell culture incubator. After 2 weeks, the cell colonies were washed 3 times with 1 × PBS. Colonies were fixed with 4% paraformaldehyde for 30 min and stained with 0.1% crystal violet (Solarbio, China) for 30 min.

**Wound-healing assay**

THCA cells were seeded into 6-well plated and scratched with a pipette tip after an overnight incubation. Then, wash off the detached cells with PBS and continue incubating in serum-free medium.

**Transwell migration and invasion assays**

Chamber invasion ability were performed with Corning chambers (Corning, USA). 1×105 cells cultured with 200 µl serum-free media were seeded onto Transwell chambers. After incubating for 24 hours, cells were fixed with 4% polymethanol for 20 minutes, then stained with 0.1% crystal violet for 15 minutes, and pictures were taken under an inverted microscope for statistics.

**Animal studies**

Animal experiments were conducted under the guidelines of the Laboratory Animal Center of Wuhan University of Science and Technology. BALB/c Nude mice, which were 4 weeks old and ~15 g weight per mouse were purchased from Beijing Huafukang Experimental Animal Co., Ltd., and housed in the Experimental Animal Center of Wuhan University of Science and Technology. The cell suspension 2x10^6^ cells/ml was injected subcutaneously into the dorsal side of the Nude mice. The mice were sacrificed 4 weeks later for biochemical and histopathological analyses were performed on the tumor samples**.**

**Immunohistochemistry**

After formalin fixation and paraffin embedding, the tissues were deparaffinized in xylene and rehydrated in graded ethanol. For immunohistochemistry, the slides were immersed in 3% hydrogen peroxide and incubated with primary Abs overnight. The Abs included anti-PPP4R1 (Thermo, PA5-76370, 1:50 dilution), anti-VEGFR2(26415-1-AP, 1：1000， Proteintec), p-VEGFR2 (abclonal, AP0382, 1:200 dilution) and CD31 (Servicebio, GB11063-2, 1:200 dilution). Subsequently, the slides were incubated with HRP-conjugated secondary Abs (Santa Cruz Biotechnology, sc-2357, sc-516102). Finally, after the application of DAB chromogen, tissue sections were stained with hematoxylins.

**Statistical analysis**

The data analyses were carried out with using GraphPad Prism 8 software. Data are presented as the mean ± SD, and repeat all experiments three times. For all analyses, a P-value less than 0.05 from a two-tailed test was considered statistically significant.
