# Supplementary figures and images for: lncRNA IGF2‐AS regulates miR‐500a‐3p/PPP4R1/p‐VEGFR2 signalling pathway to promote thyroid carcinoma progression and tubulogenesis
Source: Clin Transl Med. 2023 Apr 17;13(4):e1240. doi: 10.1002/ctm2.1240 (PMC10111635; doi:10.1002/ctm2.1240)

**Fig.1I**


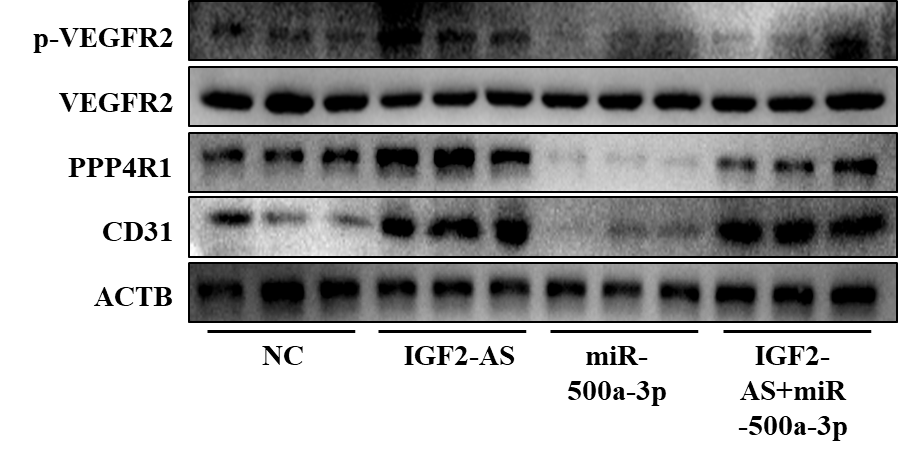


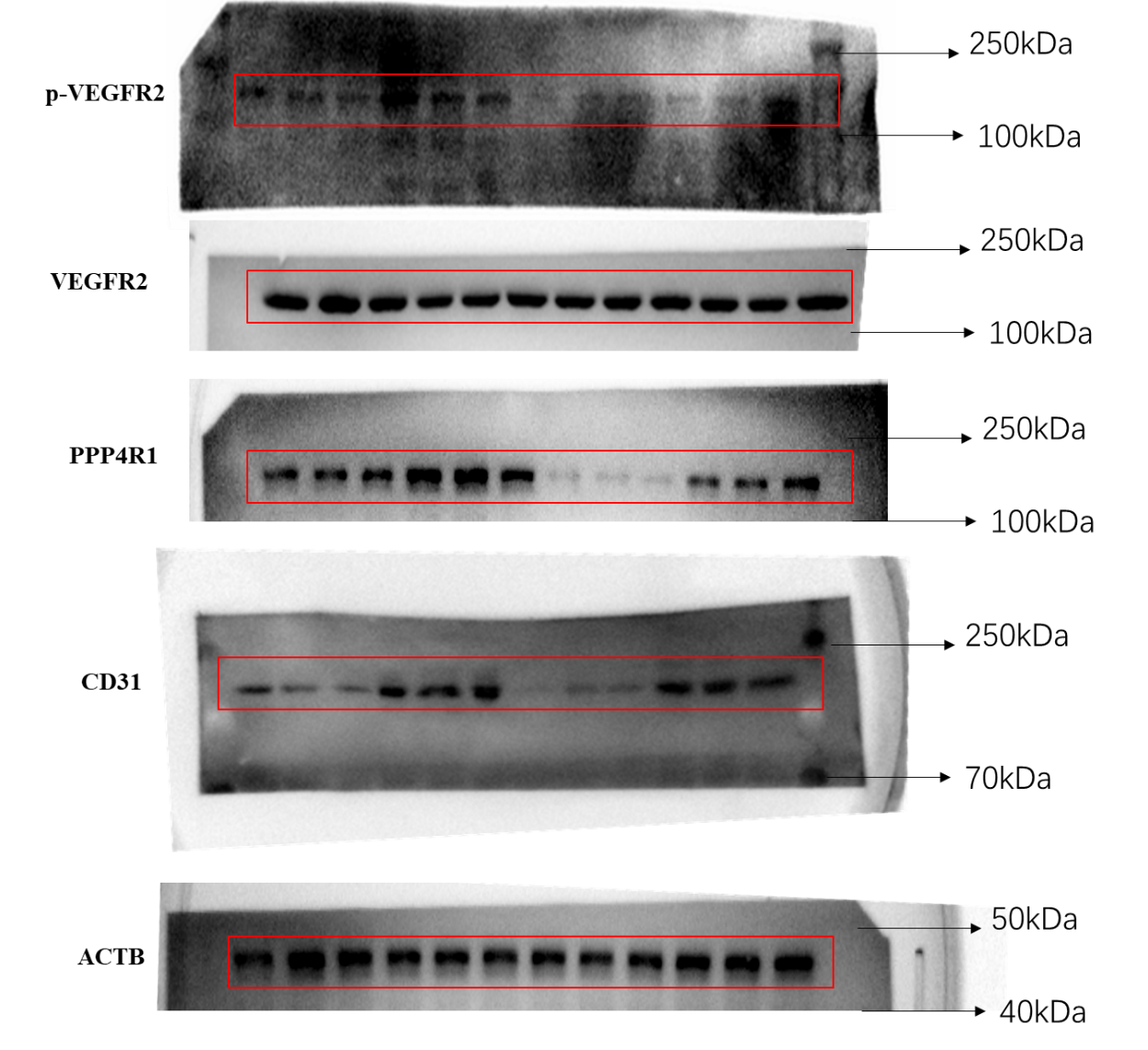


**Fig.S8F**


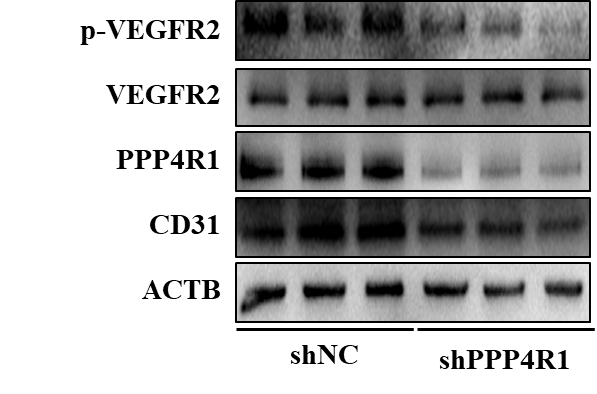


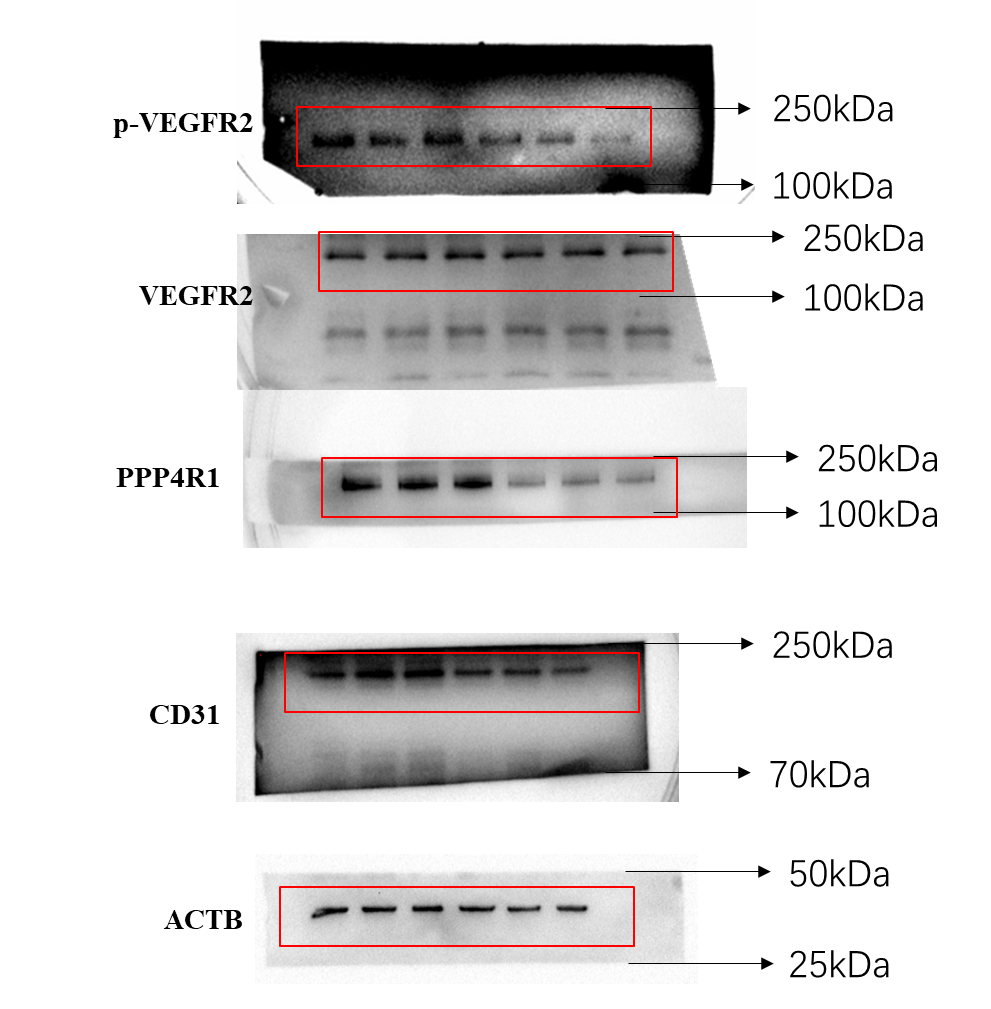


**Fig.S9C**


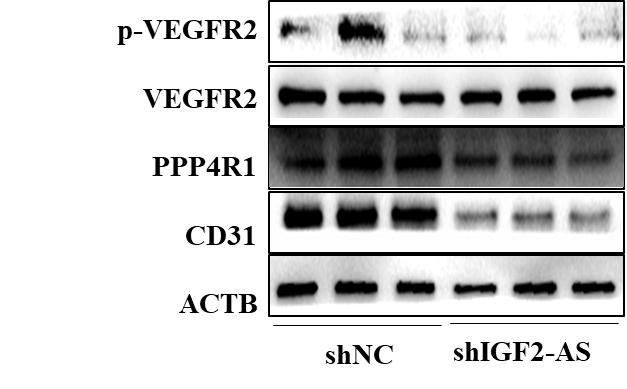


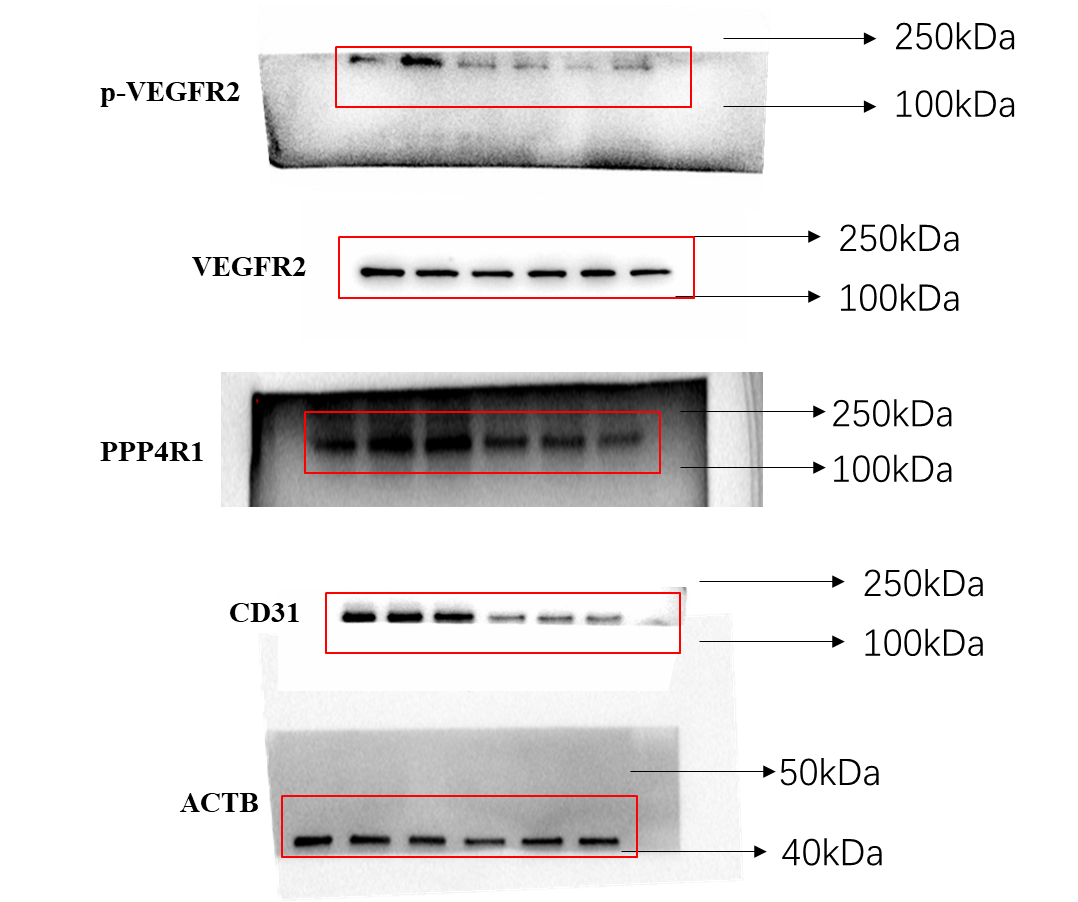


**Fig.S7C, Fig.S9A, Fig.S9B**


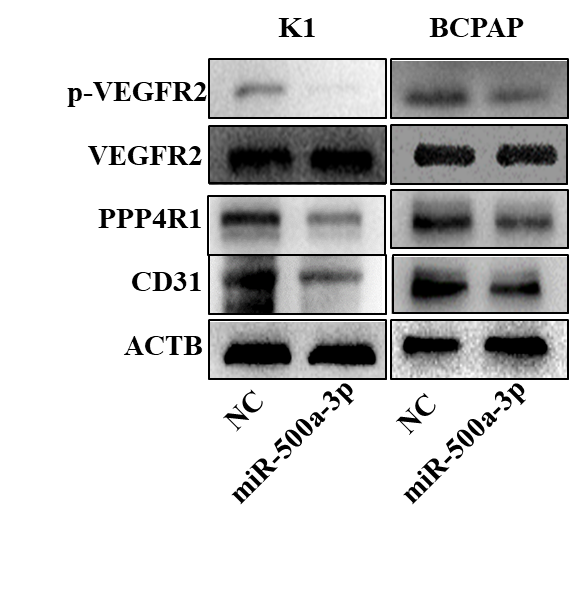

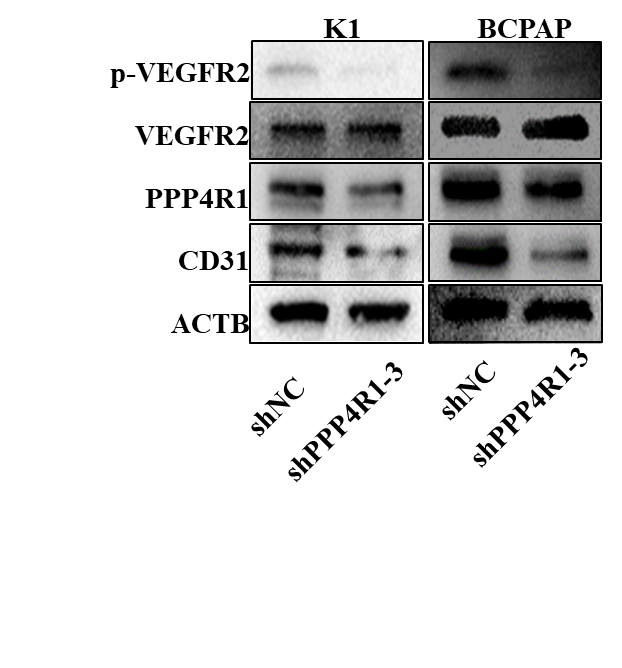

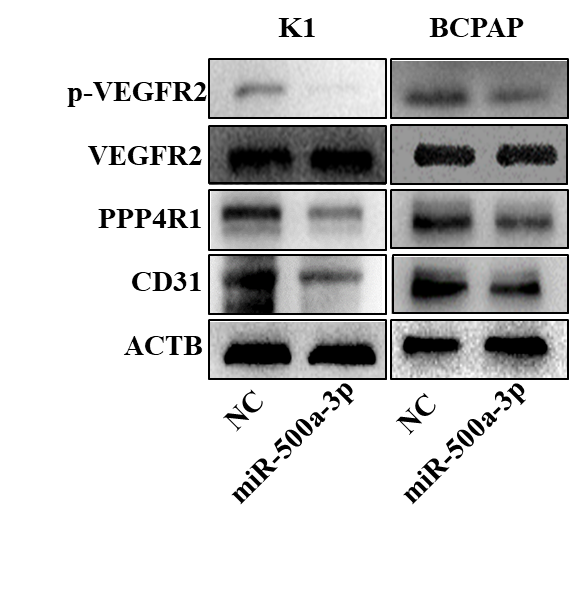


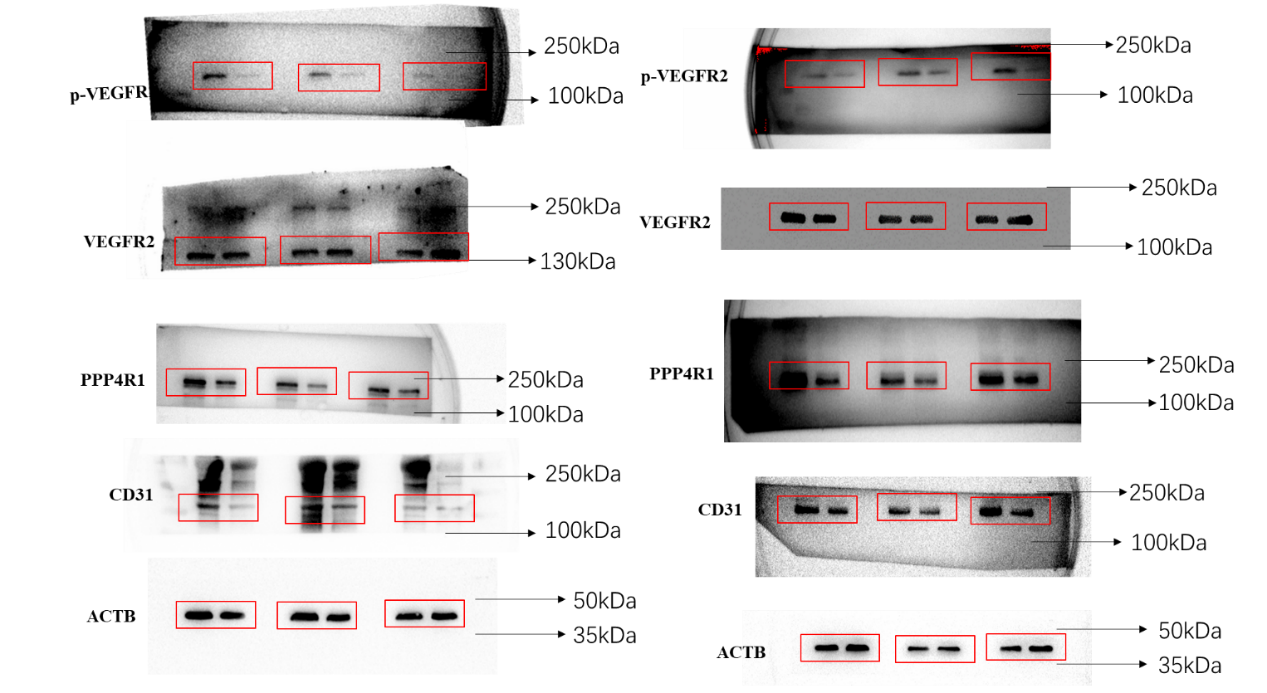

Supplement: Supplementary file 3 — Supporting Information [file CTM2-13-e1240-s011.docx]

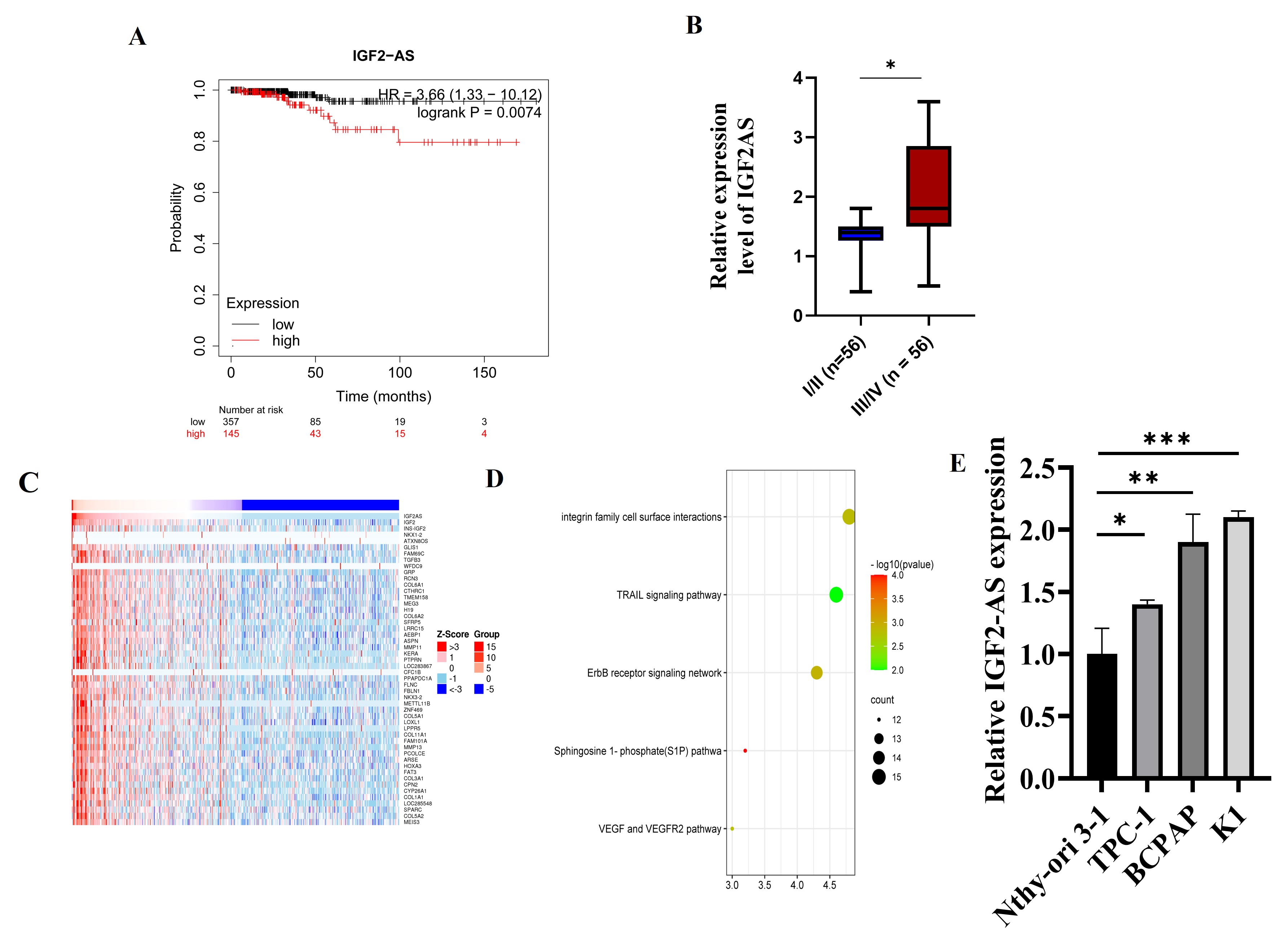

Supplement: Supplementary file 5 — Supporting Information [file CTM2-13-e1240-s007.jpg]

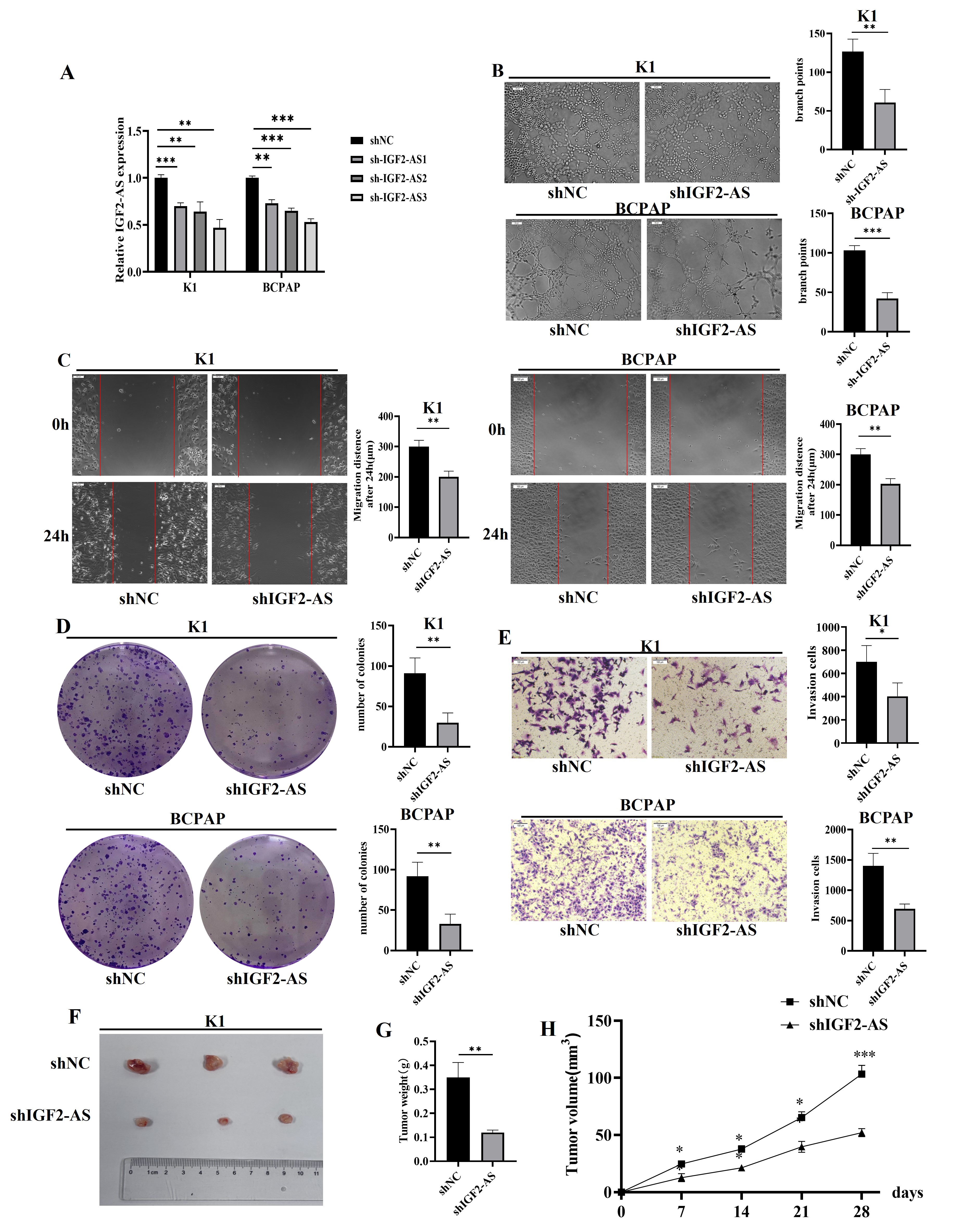

Supplement: Supplementary file 6 — Supporting Information [file CTM2-13-e1240-s006.jpg]

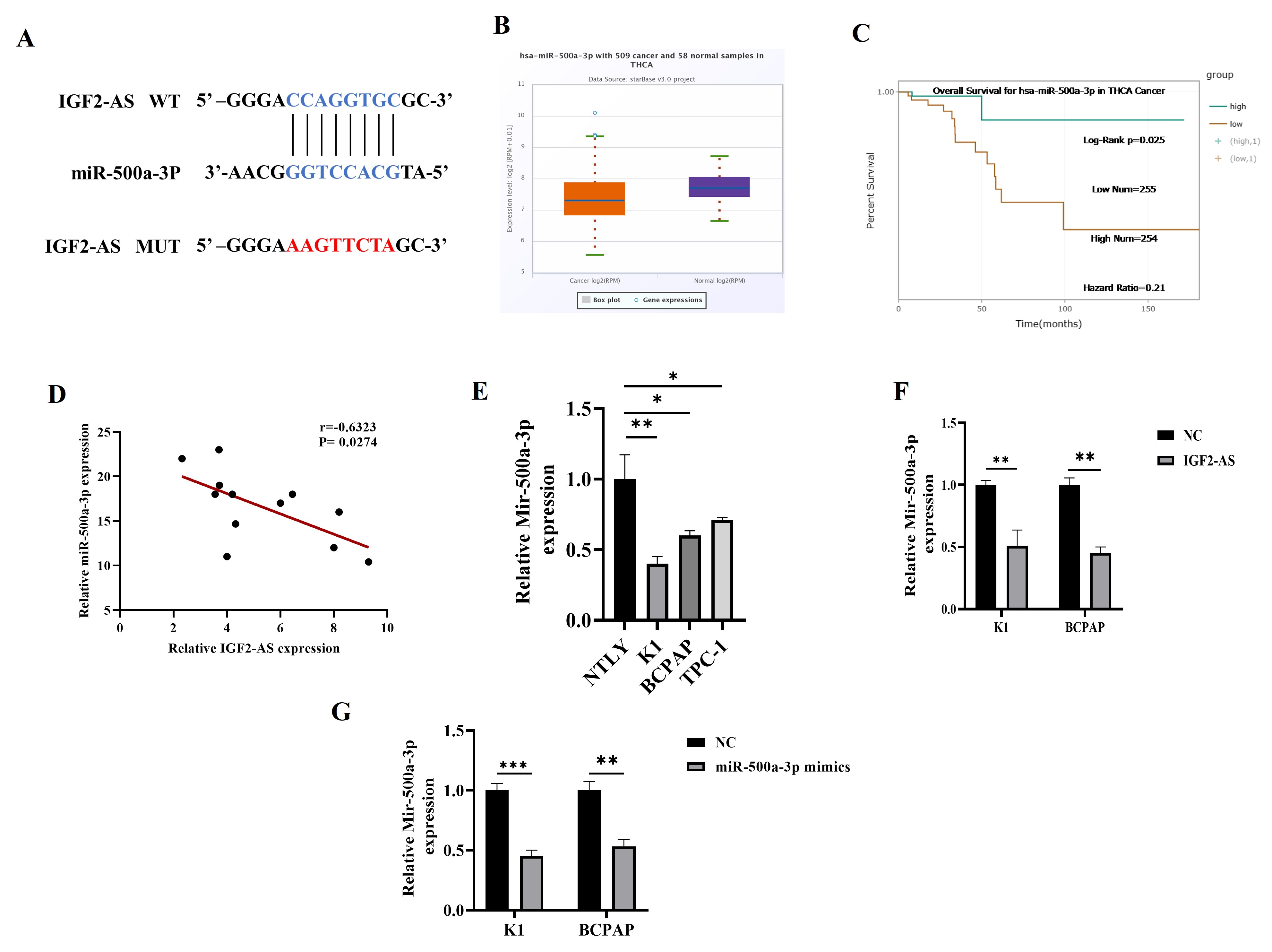

Supplement: Supplementary file 7 — Supporting Information [file CTM2-13-e1240-s012.jpg]

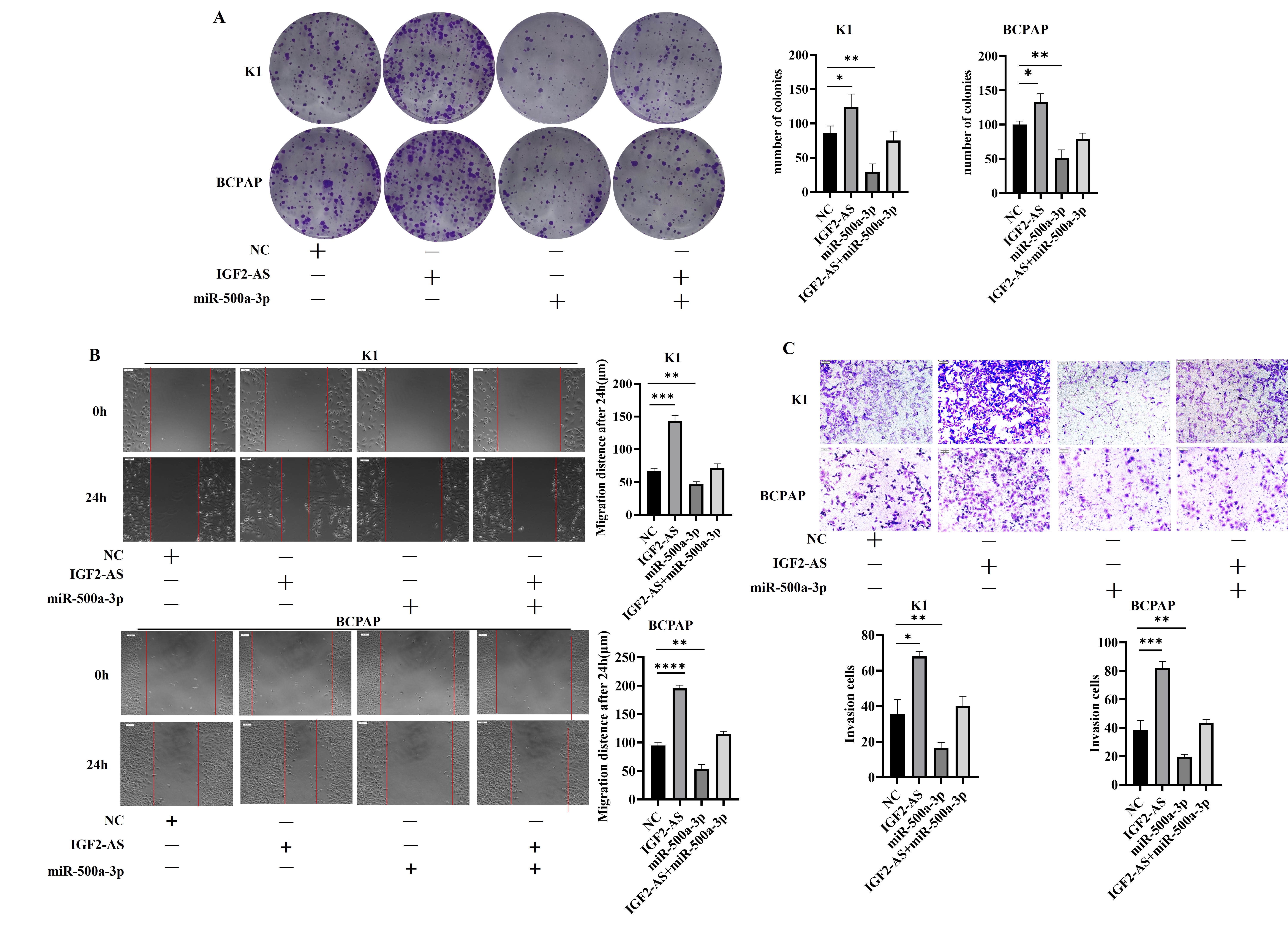

Supplement: Supplementary file 8 — Supporting Information [file CTM2-13-e1240-s001.jpg]

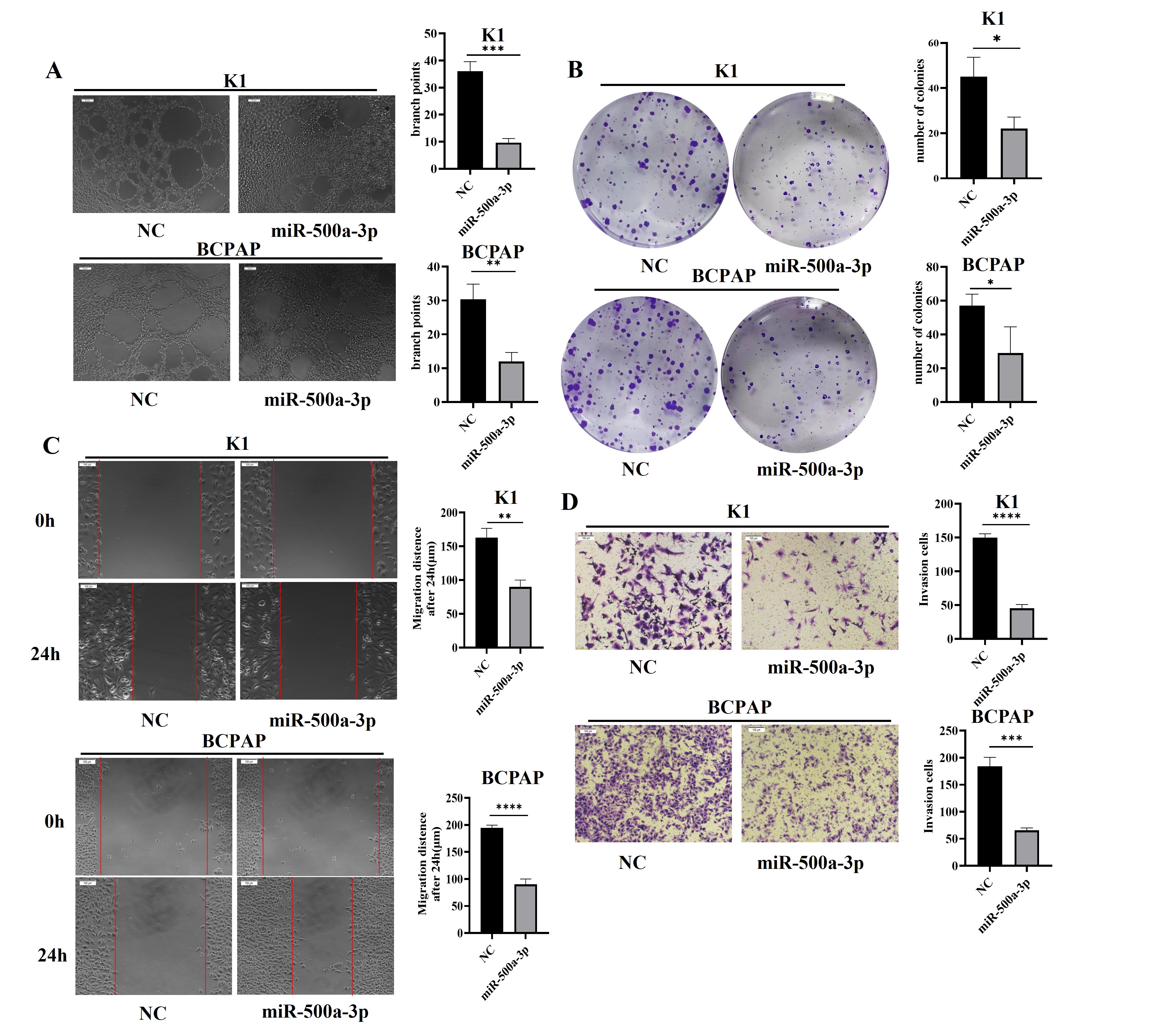

Supplement: Supplementary file 9 — Supporting Information [file CTM2-13-e1240-s009.jpg]

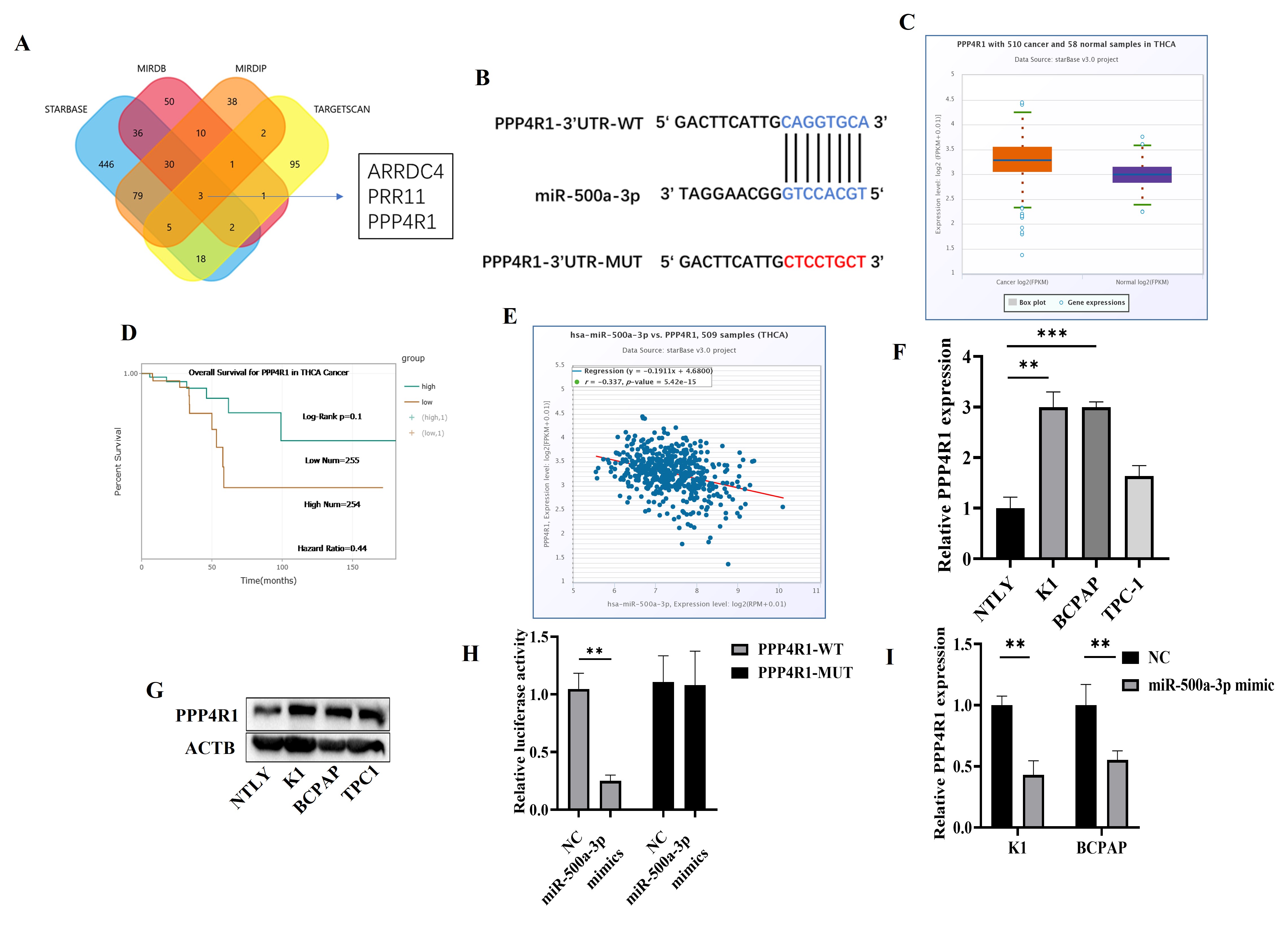

Supplement: Supplementary file 10 — Supporting Information [file CTM2-13-e1240-s013.jpg]

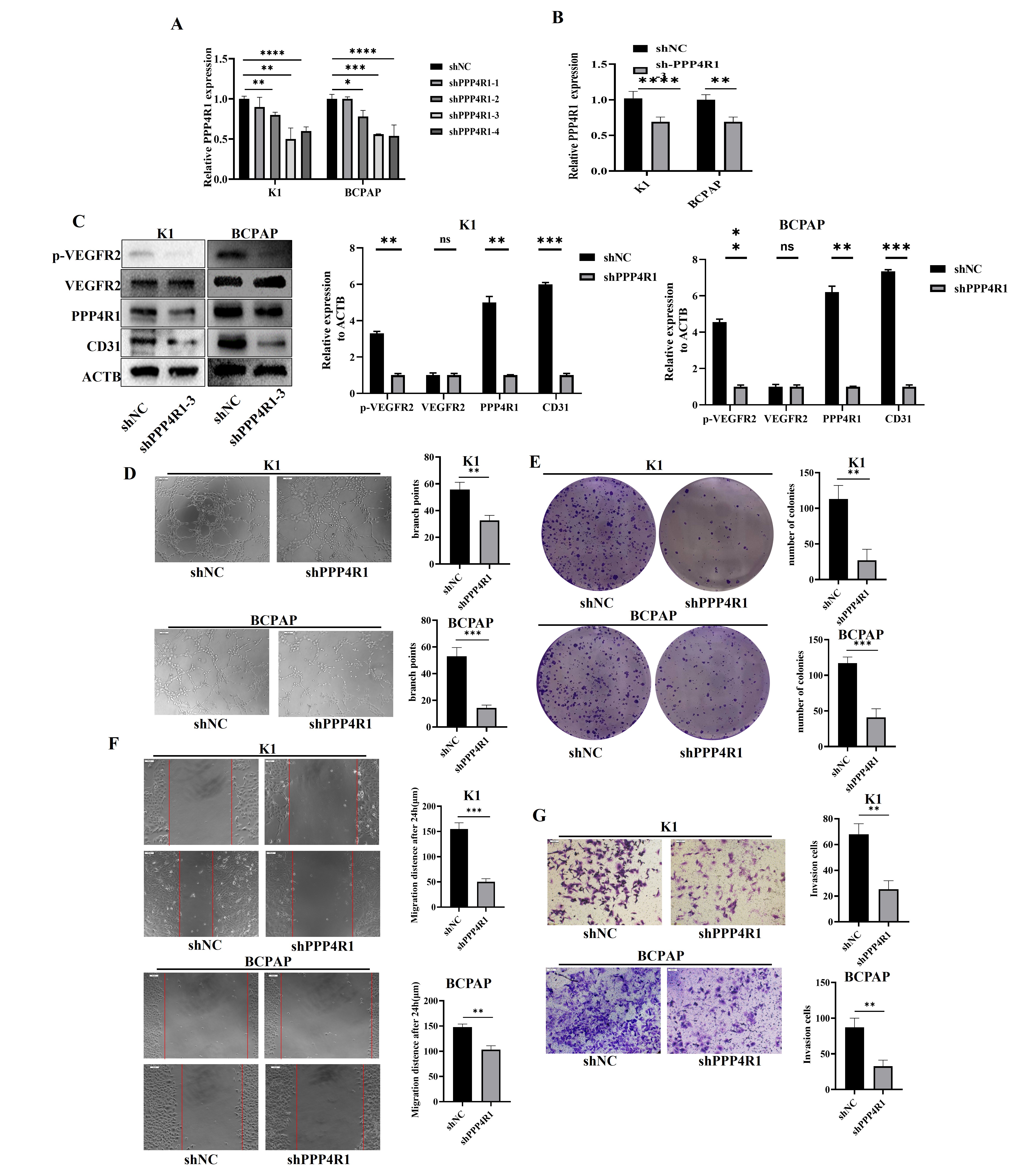

Supplement: Supplementary file 11 — Supporting Information [file CTM2-13-e1240-s004.jpg]

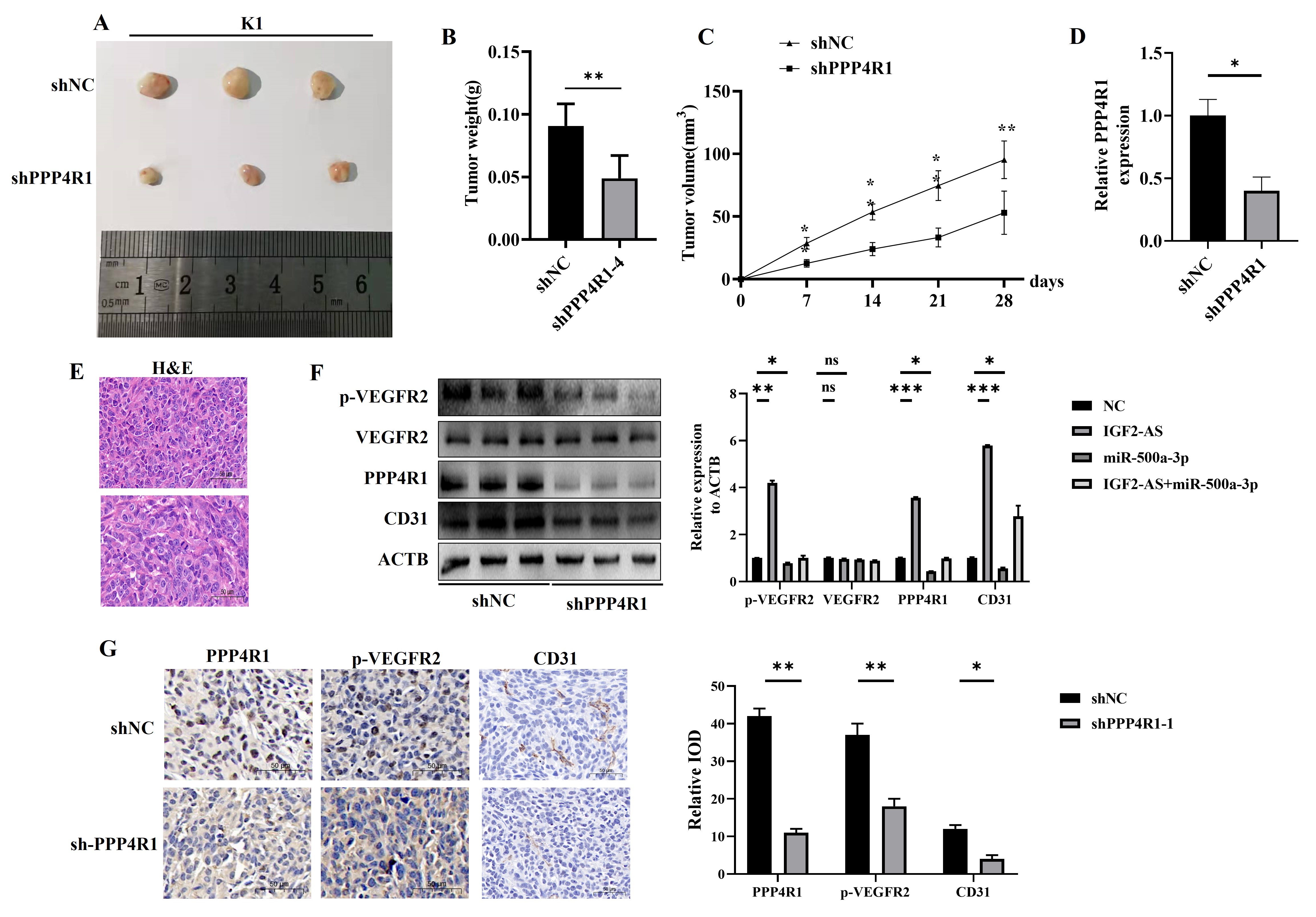

Supplement: Supplementary file 12 — Supporting Information [file CTM2-13-e1240-s010.jpg]

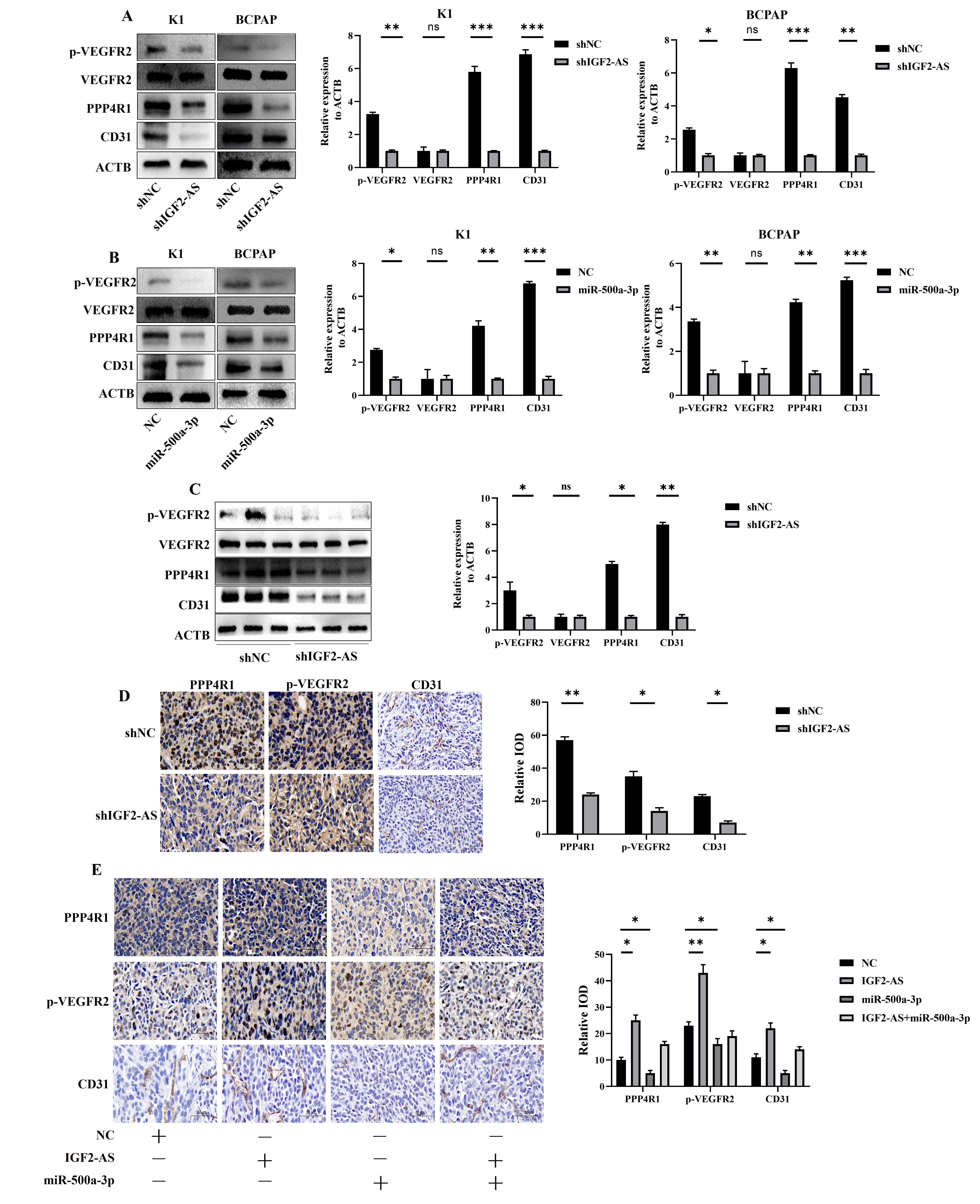

Supplement: Supplementary file 13 — Supporting Information [file CTM2-13-e1240-s002.jpg]
