# Supplementary material for: lncRNA IGF2‐AS regulates miR‐500a‐3p/PPP4R1/p‐VEGFR2 signalling pathway to promote thyroid carcinoma progression and tubulogenesis
Source: Clin Transl Med. 2023 Apr 17;13(4):e1240. doi: 10.1002/ctm2.1240 (PMC10111635; doi:10.1002/ctm2.1240)
Supplement: Supplementary file 4 — Supporting Information [file CTM2-13-e1240-s005.docx]

**Graphical Abstract**


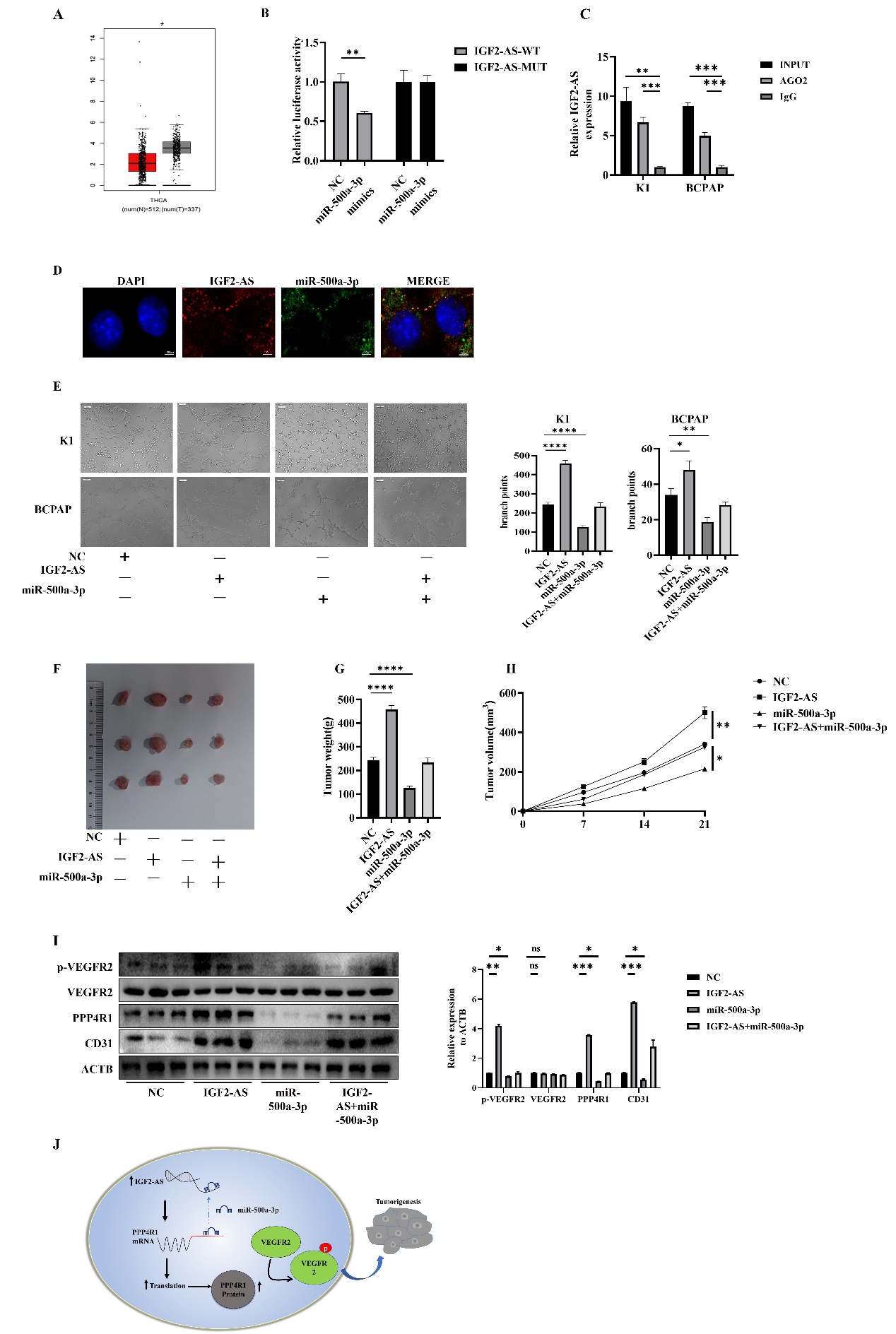


**Graphical Headlights**

1. Upregulation of LncRNA IGF2-AS expression in human thyroid cancer cells
2. LncRNA IGF2-AS sponge adsorbed miR-500a-3p
3. LncRNA IGF2-AS regulates PPP4R1 expression levels by sponge-adsorption of miR-500a-3p
